# An Emulation of Randomized Trials of Administrating Benzodiazepines in PTSD Patients for Outcomes of Suicide-Related Events

**DOI:** 10.3390/jcm9113492

**Published:** 2020-10-29

**Authors:** Michael Gilbert, Andrew Dinh La, Noah Romulo Delapaz, William Kenneth Hor, Peihao Fan, Xiguang Qi, Xiaojiang Guo, Jian Ying, Lirong Wang

**Affiliations:** 1 School of Pharmacy, University of Pittsburgh, Pittsburgh, PA 15206, USA; mig64@pitt.edu (M.G.); adl64@pitt.edu (A.D.L.); nrd33@pitt.edu (N.R.D.); wkh7@pitt.edu (W.K.H.); 2 Department of Pharmaceutical Sciences, Computational Chemical Genomics Screening Center, University of Pittsburgh School of Pharmacy, Pittsburgh, PA 15206, USA; PEF14@pitt.edu (P.F.); xiq24@pitt.edu (X.Q.); xig53@pitt.edu (X.G.); 3 Department of Internal Medicine, University of Utah, Salt Lake City, UT 84132, USA; jian.ying@hsc.utah.edu

**Keywords:** PTSD, benzodiazepine, suicide, suicide-related behaviors, clinical trial emulation

## Abstract

Benzodiazepines is a class of medications frequently prescribed to patients with post-traumatic stress disorder. Patients with PTSD have a notable increased risk of suicide compared to the general population. These medications have been theorized to increase suicidality and pose a risk when used in this patient population. Previous research has found little utility of using benzodiazepines in the PTSD population. However, benzodiazepines are still commonly prescribed by some clinicians for their symptomatic benefit. This study aims to identify the comparative efficacy of commonly prescribed benzodiazepines including midazolam, lorazepam, alprazolam, clonazepam, diazepam and temazepam in relation to suicide-related behaviors (SRBs). A total of 38,807 patients who had an ICD9 or ICD10 diagnosis of PTSD from January 2004 to October 2019 were identified through an electronic medical record database. Inclusion criteria include patients that initiated one of the above benzodiazepines after PTSD diagnosis. Exclusion criteria include previous history of benzodiazepine usage or history of SRBs within the last year prior to enrollment. For patients enrolled in this study, other concomitant drugs were not limited. The primary outcome was onset of SRBs with each respective benzodiazepine. SRBs were identified as ideation, attempt, or death from suicide. We emulated clinical trials of head-to-head comparison between two drugs by pooled logistic regression methods with the Firth option adjusting for baseline characteristics and post-baseline confounders. A total of 5753 patients were eligible for this study, with an average follow up of 5.82 months. The overall incidence for SRB was 1.51% (87/5753). Head-to-head comparisons identified that patients who received alprazolam had fewer SRBs compared to clonazepam (*p* = 0.0351) and lorazepam (*p* = 0.0373), and patients taking midazolam experienced fewer relative incidences of SRBs when compared to lorazepam (*p* = 0.0021) and clonazepam (*p* = 0.0297). After adjusting for the false discovery rate (FDR), midazolam still had fewer SRBs compared to lorazepam (FDR-adjusted *p* value = 0.0315). Certain benzodiazepines may provide a reduced risk of development of SRBs, suggesting careful consideration when prescribing benzodiazepines to the PTSD population.

## 1. Introduction

### 1.1. Background PTSD, SRB and Benzodiazepines

Post-traumatic stress disorder (PTSD) is a mental disorder characterized by psychological and behavioral disturbances that are developed after a person is exposed to traumatic events. The DSM-5 criteria diagnose PTSD if related symptoms last for over a month. Symptoms of PTSD include the experience of trauma (through intrusive thoughts, nightmares, or flashbacks), avoidance of certain situations or environments, hypervigilance, and hyperarousability [1].

Patients diagnosed with PTSD often have comorbid Diagnostic and Statistical Manual (DSM) disorders. Frequently associated disorders include generalized anxiety disorder, major depressive episodes, and substance abuse [2,3]. In addition, patients diagnosed with PTSD have a potentially increased risk of suicide ideation and attempt. A study conducted by Sareen and colleagues found that PTSD alone out of five other anxiety-related disorders (social phobia, panic disorder, agoraphobia, generalized anxiety disorder, and simple phobia) was associated with a significant increase in suicide ideation and attempts [4,5].

Suicide-related behaviors (SRBs) are defined as the act of ideation, attempt, and/or death from suicide. Certain mental disorders such as depression have shown to be a risk factor of death by suicide [6]. It is not clear cut whether PTSD itself puts an individual at a greater risk of SRBs. A meta-analysis of 50 studies that focused on the correlation of PTSD and suicidal ideation found no association between the two [7]. Separate smaller studies have shown that those diagnosed with PTSD had a greater rate of suicide than those not diagnosed with PTSD [8].

Treatment for PTSD includes behavioral therapy such as exposure therapy and cognitive restructuring [9]. Pharmacotherapy is also commonly utilized to control symptoms related to PTSD. Commonly prescribed medications include antidepressants to decrease flashbacks and nightmares, antianxiety to reduce hyperarousability and sleep aids to reduce symptoms of restlessness [10,11]. Frequently utilized selective serotonin reuptake inhibitors (SSRIs) include sertraline, paroxetine, and fluoxetine. Venlafaxine, a serotonin and norepinephrine reuptake inhibitor (SNRI), is also commonly prescribed [12]. Less commonly used medications for PTSD associated symptoms include antipsychotics such as quetiapine, olanzapine, and risperidone [13]. In addition, prazosin, an α1-blocker has been reported to be significantly more efficacious than placebo in reducing distressing dreams in PTSD patients [14].

Benzodiazepines are a class of medications that are commonly prescribed in PTSD patients. Commonly prescribed benzodiazepines include alprazolam, lorazepam, and clonazepam. Benzodiazepines have several therapeutic benefits, such as sedative, antianxiety, and anticonvulsant activities due to an increase in GABA [15,16]. While benzodiazepines are commonly prescribed for PTSD or related comorbidities such as anxiety and insomnia, recent literature suggests a lack of efficacy for PTSD-related symptoms. Guina and colleagues conducted a meta-analysis of clinical trials and observational studies and discovered a lack of efficacy for benzodiazepines in the PTSD population and the potential for worsening the prognosis and severity of PTSD. In addition to worsened PTSD outcomes, several studies within the meta-analysis showed those who took benzodiazepines had an increased association of experiencing depression, anxiety, and aggression [17]. 

### 1.2. What Is Known about the Relationship between Benzodiazepines and SRB in the PTSD Population

Benzodiazepines are one of the most common medications prescribed for PTSD patients. Between 30% and 74% of PTSD patients have been prescribed a benzodiazepine in the past [18]. While many practice guidelines recommend against the use of benzodiazepines in the PTSD population, some clinicians still prescribe them for symptomatic benefits [19]. In addition, benzodiazepines have been associated with an increase in suicide-related behaviors including ideation, attempts, and completed suicide [9,17,20].

### 1.3. What Is Unknown about Benzodiazepines and SRB in the PTSD Population 

Because the use of benzodiazepines is still so prevalent among the PTSD population, despite concerns about their use from practice guidelines, we believe that it is crucial to find trends among the class that minimize exacerbations of suicide-related behaviors. Currently, there are few data on studies that examine whether specific benzodiazepines have a decreased risk of SRBs compared to other benzodiazepines. We aim to examine the prevalence of suicide-related behaviors in PTSD patients among patients that have been prescribed benzodiazepines. The goal of this study is to identify whether specific benzodiazepines have a decreased risk of causing suicide-related behaviors, which we define as ideation, attempted, or completed suicide.

## 2. Material and Methods

### 2.1. Study Population

We accessed the Neptune system, which manages patient electronic medical records from January 2004 to October 2019, at the University of Pittsburgh. The database includes demographic information, diagnoses, encounters, medication prescriptions, prescription fill history, and laboratory tests. 

### 2.2. Inclusion/Exclusion Criteria and Endpoints/Follow Up

The baseline inclusion criteria include initial benzodiazepine after the diagnosis of PTSD (qualifying event), no history of suicide-related events within one year prior to the enrollment, no prior use of benzodiazepines within one year, and at least one year of history in the electronic medical record to ensure that patients can be assessed for the inclusion. If eligibility criteria were met, patients were followed to the onset of the first suicide-related event (primary outcome) or until a loss to follow up (stopping the use of the benzodiazepine of interest, initiating or switching to another benzodiazepine, patients’ data being no longer accessible or reaching the end time of this study). Exclusion criteria include previous history of benzodiazepine usage or history of SRBs within the last year prior to enrollment. Patients included in this study could take any other medications for the treatment of their comorbidities. 

### 2.3. Background on Emulating Randomized Controlled Trial 

Creating guidance for clinical guidelines requires sound evidence on the effects of their recommendations on relevant outcomes. The gold standard of investigation relies heavily on randomized controlled trials. However, randomized controlled trials are not always feasible often due to money, time, or ethical constraints. Observational data are often used as an alternative to randomized controlled trials. 

Studies using observational data are often subject to selection and immortal-time bias, which can be controlled in randomized controlled trials. Emulation of a randomized controlled trial is a technique used to control these two biases. Emulation attempts to eliminate selection bias by specifying a target trial and creating an appropriate protocol. This allows for the comparison of individuals newly initiated on the medication against those who were never initiated. Immortal-time bias can be minimized by analyzing the data so that time of eligibility for treatment and time when treatment is initiated is the same [21].

### 2.4. Target Trials

In order to be included in the trial, eligible PTSD patients must have no experience of SRBs and no usage of any benzodiazepine during one year prior to the enrollment. Patients were randomly assigned to one of the compared benzodiazepine arms in the target trial. Benzodiazepines of interest include alprazolam, clonazepam, diazepam, lorazepam, midazolam, and temazepam. Patients could take any other medications for the treatment of their comorbidities. The trial will be stopped if a patient stops using the drug assigned or switches or starts using another benzodiazepine, loses follow up, or experiences SRBs. The outcome evaluated is the event of SRBs.

### 2.5. Emulating Target Trials

We attempted to emulate randomized controlled trials similar to the work of Danaei and colleagues [22]. Confounding variables had to be measured at least once during the study duration. These variables can be accessed below in Table 1 of the Methods section and categories are based on the ICD9 and ICD10 codes described in Appendix A. In addition to the baseline eligibility criteria that are discussed above, participants needed one or more years of continuous recording in the UPMC medical records and at least one medical visit within one year of initiation of the trial. Monthly trials were collected from the UPMC EMR database from January 2004 to October 2019 (190 monthly intervals). Patients could be included in this study multiple times if a one-year washout period of benzodiazepine was fulfilled and all inclusion criteria were met. Those eligible were placed in a specific target trial arm based on the benzodiazepine used. The target trials of benzodiazepines were then compared, with the primary outcome being SRBs experienced. Study duration was stopped if the medication of interest was discontinued or if the patient’s EMR data were no longer available (loss to follow up or death).

### 2.6. Per-Protocol Analysis

A per-protocol analysis was utilized which requires that all the patients fully completed the given protocol regime. The effect of the two drugs is compared to the outcomes of two cohorts who completed the treatment originally allocated. This analysis may give rise to bias by baseline confounders and post-baseline, time-varying confounders. An approach suggested by Danaei and colleagues used a pooled logistic regression model in order to estimate the effect of treatment [22]. They used inverse probability weighting in order to create a population where treatment is independent of prognostic factors history. The baseline information included 12 categories of mental disorders, age, gender, and number of emergency department visits within one year prior to the enrollment which can be found in Table 1. These baseline variables were adjusted in the pooled logistic regression models. To test the effects of concomitant medications, we performed an additional analysis by adjusting the most frequently used drugs of the central nervous system in drug pair(s) of interest.

In our emulation, we only fit a logistic regression model to adjust the censoring effects and did not consider the switch from one of the paired benzodiazepines to the other one since we believe this situation can be modeled by the censored model as well. 

In addition, we used robust variances to calculate conservative 95% confidence intervals and also truncated the inverse-probability weights to their 99th percentile. Those are options provided by the code from www.hsph.harvard.edu/causal/software. We also applied the Firth option in the logistic regression to accommodate the presence of rare events or complete separation. The hazard ratio was calculated from the treatment effect estimate of logistic regression. Data sets were prepared by Python and the final analyses were carried out using SAS 9.4 (SAS Institute Inc., Cary, NC, USA). The hazard ratio was calculated from the treatment effect estimate of logistic regression. We used the false discovery rate (FDR) to control the type I error caused by multiple testing. The FDR q value is calculated by R [23] base package function “p.adjust”.

## 3. Results

**Figure 1:**
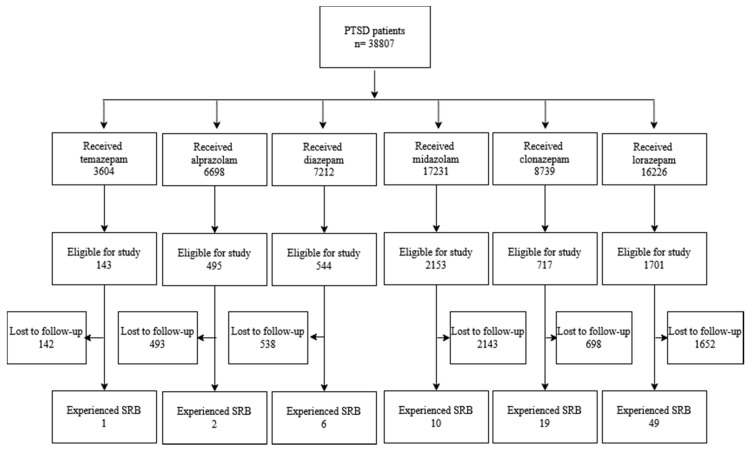
Selection process for emulation.

**Figure 2:**
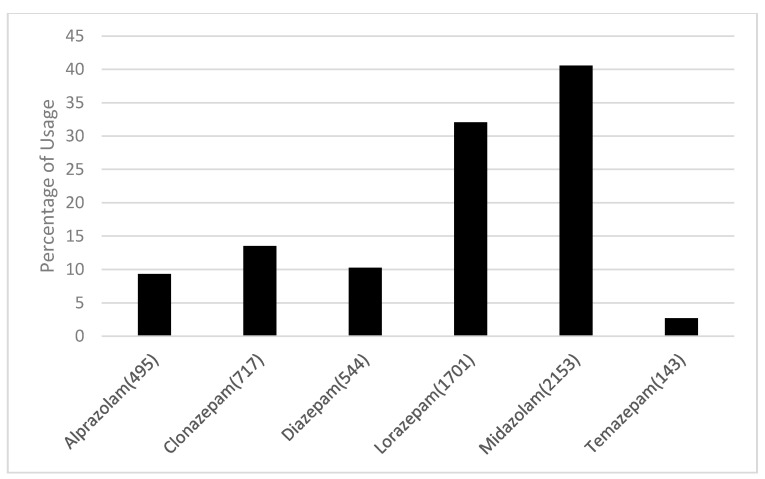
Percent of eligible patients with PTSD receiving a certain type of benzodiazepine.

A total of 38,807 patients diagnosed with PTSD were identified. After application of our inclusion and exclusion criteria, 5753 patients were eligible for this study. Baseline characteristics are shown in Table 1. The emulation process is shown in Figure 1. Percent usage for each benzodiazepine is shown in Figure 2. Table 2 displays the average follow up time for each treatment.

Elaboration of categories can be found in Appendix A. A list of concomitant antidepressants can be found in Appendix C.

Of the 5753 patients eligible for this study, the average follow-up time was 5.84 months and 87 events happened. Outcomes were adjusted for baseline characteristics and concomitant therapy for antidepressant usage. While on benzodiazepine therapy, 60.7 to 79.6% of patients were taking an antidepressant at the same time. The overall incidence for SRBs was 1.51% (87/5753). The incidence of SRBs for each arm was: midazolam 0.046% (10/2153), lorazepam 2.88% (49/1701), alprazolam 0.04 % (2/495), clonazepam 2.65% (17/717), diazepam 1.10% (6/544), and temazepam 0.07% (1/143).

As shown in Table 3, head-to-head comparisons are conducted for every pair of benzodiazepines to compare their relative efficacies in the management of SRBs. Notable findings included patients who received alprazolam experienced statistically significantly fewer SRBs compared to clonazepam (*p* = 0.0351) and lorazepam (*p* = 0.0373). Patients taking midazolam experienced fewer relative incidences of SRBs when compared to lorazepam (*p* = 0.0021) and clonazepam (*p* = 0.0297). After adjusting for the false discovery rate (FDR), midazolam still had fewer SRBs compared to lorazepam (FDR-adjusted *p* value = 0.0315).

**Figure 3:**
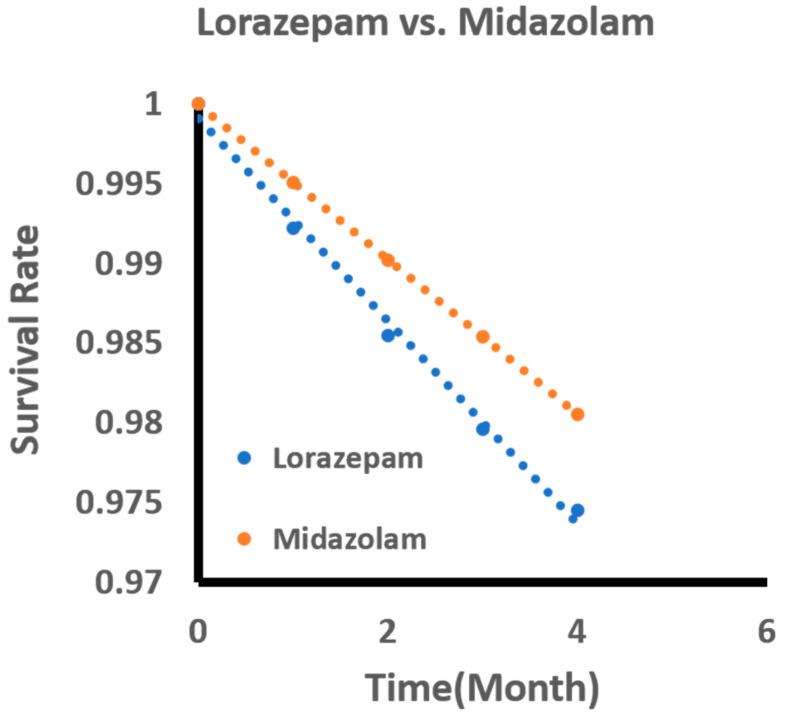
Standardized survival curve based on head-to-head comparisons of lorazepam and midazolam. The curve shows significant differences in survival rates compared between lorazepam and midazolam.

Figure 3 showed the standardized survival curve for lorazepam vs. midazolam. The resulting curve was the outcome of adjusting covariates and may not accurately reflect the SRBs listed in the head-to-head comparison. The resulting curve was generated by following the work by Danei and colleagues [22]. Each survival curve was estimated by using the parameters from the pooled logistic model.

To assess the effects of using concomitant medications on the SRBs, we also adjusted the most used medications. Those concomitant drugs have been used at the baseline time in more than 5% of users of a benzodiazepine drug of interest. After adjusting those drugs, the hazard ratio of lorazepam and midazolam is 2.56 (1.15, 5.69), with a *p* value of 0.0209 (Appendix D, the ‘treatment’ variable where the hazard ratio and confidence interval were in logarithmic scale). It is not a surprise that antipsychotics such as haloperidol and aripiprazole are also significantly associated with an increased risk of SRBs. Interestingly, thiamine is associated with a decreased risk of SRBs. We should be cautious with result interpretation as the use of those medications might also indicate the comorbidities we have adjusted for. Detailed information on all the concomitant medications can be found in Appendix E.

## 4. Discussion

Our analysis was based on electronic medical records from the Neptune system at the University of Pittsburgh. The findings of this study hope to provide insight into whether certain benzodiazepines have a decreased risk of SRBs in the PTSD population. Statistically significant results were found with our comparison of benzodiazepines. Midazolam, a benzodiazepine frequently used for its anesthetic and sedative properties in the perioperative setting, showed a statistically significant decrease in SRBs when compared to lorazepam after adjusting for FDR. It should be noted that midazolam was the most frequently utilized benzodiazepine, with 37% of eligible participants taking this medication. While midazolam has therapeutic uses for acute relief of seizures, it is highly dispensed in the emergency department for preoperative sedation, which may provide an explanation for its high population percentage in this study. 

The findings of this study indicate that commonly used benzodiazepines such as lorazepam may put individuals at an increased risk of SRBs. The results of this study identify the need for special consideration when prescribing benzodiazepines, especially in populations that are at an increased risk for suicide. 

Before adjusting for FDR, head-to-head comparisons identified several significant trends. Patients who received alprazolam recorded fewer SRBs compared to clonazepam (*p* = 0.0351) and lorazepam (*p* = 0.0373). Furthermore, those taking midazolam experienced fewer relative incidences of SRBs when compared to lorazepam (*p* = 0.0021) and clonazepam (*p* = 0.0297). Since the number of eligible patients for this study was relatively small and few patients recorded experiencing SRBs, more data will be needed to validate these results. 

These findings are particularly applicable in a clinical setting when having to choose between benzodiazepines for a specific indication. Benzodiazepines have a variety of different FDA-approved indications ranging from anxiety to insomnia. For instance, lorazepam and alprazolam are both FDA approved for anxiety; however, alprazolam showed significantly fewer SRBs when compared to lorazepam, before adjusting for FDR [24]. With little previous research in the PTSD population specific to SRBs and benzodiazepines, the results from our study may hopefully be utilized to guide patient-specific pharmacotherapy recommendations. 

Previous literature analyzing the prevalence of SRBs with benzodiazepines is limited. Few studies have compared the relationship of SRBs within the benzodiazepine class. Dodds performed a meta-analysis of 17 studies to examine whether benzodiazepines increase the risk of suicide. The analysis identified that a majority of the studies included showed an increase in the risk of suicide, which may be mediated by an increase in aggression [24]. Concerning the PTSD population, the meta-analysis by Guinea and colleagues discovered that benzodiazepines may worsen the severity of PTSD symptoms that include increases in depression, aggression, and substance abuse [17]. Considering the prevalence of benzodiazepine usage within the PTSD population, it is pertinent to expand upon this previous research and provide analytics within the benzodiazepine class to guide specific pharmacotherapy recommendations. 

With the current lack of research on this topic, we hope that our study provides a gateway to discovering the relationship between different drugs and SRBs within this population. When choosing the appropriate drug therapy for a patient, it is important to weigh the risks and benefits of a medication. While benzodiazepines are not approved for PTSD-related symptoms, some clinicians believe that their symptomatic benefits outweigh the risks and prescribe them [19]. We hope that the results of this study may be used in further investigations between the relation of benzodiazepines and PTSD to minimize potential harm to these patients.

This study contains several limitations. In our model, we controlled for antidepressants only. We did not control for other medications such as atypical antipsychotics and alpha antagonists which have been hypothesized to treat PTSD [13]. While the goal was to emulate a randomized controlled trial to the best of our ability, controlling for all background therapy was unrealistic considering the complexity and variety of treatments of those with PTSD. Furthermore, comorbidities were modified based on pooled logistics from baseline information in an attempt to reduce confounding variables in this study. 

As a retrospective study, even if conducted to emulate a randomized controlled trial, the clinical presentation of SRBs was dependent on documentation within a patient’s medical record. If a health care provider was not aware of the presentation of SRBs, the most obvious culprit being ideation, they may not have recorded the event into the EMR. While a randomized controlled trial would have provided better evidence, such a trial may be unethical to conduct and most likely be infeasible. 

Additionally, our study may not have had adequate power to detect a statistically significant difference between the two groups. In our study, the overall incidence of SRBs in PTSD patients taking benzodiazepines was less than 2%. Small changes in the recorded SRB could significantly change the results of our study. Further, we did not delineate the differences within SRBs. We defined SRBs as those behaviors which included suicidal ideation, attempt, or completion. However, given the overall low incidence of SRBs within this population, it may be difficult to obtain an adequate sample size. 

## 5. Conclusions

The findings of this study are promising, and we have several future goals to expand upon this research. First, we acknowledge that the sample size of this study is small. It may be beneficial to compare our findings from the UPMC EMR records to other EMR data sets in various hospitals to validate the results. We are also interested in examining the possible molecular mechanisms of the different effects of those benzodiazepines on SRBs. In conclusion, the results of our study suggest that there needs to be careful consideration when prescribing benzodiazepines in those at an increased risk of suicide.

## Figures and Tables

**Table 1:** Level 0 represents a patient not having a diagnosis that fits under the category; level 1 represents a patient who has a diagnosis that fits under the category.

Characteristic	Level	Alprazolam	Clonazepam	Diazepam	Lorazepam	Midazolam	Temazepam	*p* Value
N		495	717	544	1701	2153	143	-
Age, years (mean (SD))		41.94 (15.13)	39.01 (14.73)	41.10 (14.18)	40.14 (16.65)	41.02 (15.37)	44.55 (15.03)	<0.001
Male (%)		130 (26.2)	212 (29.5)	178 (32.7)	602 (35.3)	752 (34.8)	57 (39.6)	<0.001
ED visits in the past 3 months (mean (SD))		0.36 (0.76)	0.41 (0.98)	0.87 (1.34)	0.78 (1.29)	0.57 (1.02)	0.47 (0.84)	<0.001
Cat1 (%)	0	462 (93.1)	651 (90.7)	500 (91.9)	1344 (78.8)	1880 (86.9)	115 (79.9)	<0.001
	1	34 (6.9)	67 (9.3)	44 (8.1)	361 (21.2)	284 (13.1)	29 (20.1)	
Cat2 (%)	0	492 (99.2)	690 (96.1)	535 (98.3)	1620 (95.0)	2076 (95.9)	142 (98.6)	<0.001
	1	4 (0.8)	28 (3.9)	9 (1.7)	85 (5.0)	88 (4.1)	2 (1.4)	
Cat3 (%)	0	255 (51.4)	350 (48.7)	307 (56.4)	634 (37.2)	759 (35.1)	68 (47.2)	<0.001
	1	241 (48.6)	368 (51.3)	237 (43.6)	1071 (62.8)	1405 (64.9)	76 (52.8)	
Cat4 (%)	0	494 (99.6)	707 (98.5)	538 (98.9)	1647 (96.6)	2140 (98.9)	144 (100.0)	<0.001
	1	2 (0.4)	11 (1.5)	6 (1.1)	58 (3.4)	24 (1.1)	0 (0.0)	
Cat5 (%)	0	311 (62.7)	466 (64.9)	416 (76.5)	969 (56.8)	1402 (64.8)	114 (79.2)	<0.001
	1	185 (37.3)	252 (35.1)	128 (23.5)	736 (43.2)	762 (35.2)	30 (20.8)	
Cat6 (%)	0	488 (98.4)	696 (96.9)	542 (99.6)	1640 (96.2)	2115 (97.7)	139 (96.5)	<0.001
	1	8 (1.6)	22 (3.1)	2 (0.4)	65 (3.8)	49 (2.3)	5 (3.5)	
Cat7 (%)	0	496 (100.0)	718 (100.0)	544 (100.0)	1702 (99.8)	2159 (99.8)	144 (100.0)	0.528
	1	0 (0.0)	0 (0.0)	0 (0.0)	3 (0.2)	5 (0.2)	0 (0.0)	
Cat8 (%)	0	493 (99.4)	716 (99.7)	544 (100.0)	1698 (99.6)	2159 (99.8)	144 (100.0)	0.448
	1	3 (0.6)	2 (0.3)	0 (0.0)	7 (0.4)	5 (0.2)	0 (0.0)	
Cat9 (%)	0	487 (98.2)	691 (96.2)	530 (97.4)	1646 (96.5)	2141 (98.9)	137 (95.1)	<0.001
	1	9 (1.8)	27 (3.8)	14 (2.6)	59 (3.5)	23 (1.1)	7 (4.9)	
Cat10 (%)	0	482 (97.2)	701 (97.6)	538 (98.9)	1646 (96.5)	2133 (98.6)	144 (100.0)	<0.001
	1	14 (2.8)	17 (2.4)	6 (1.1)	59 (3.5)	31 (1.4)	0 (0.0)	
Cat11 (%)	0	484 (97.6)	678 (94.4)	520 (95.6)	1583 (92.8)	2058 (95.1)	139 (96.5)	<0.001
	1	12 (2.4)	40 (5.6)	24 (4.4)	122 (7.2)	106 (4.9)	5 (3.5)	
Cat12 (%)	0	496 (100.0)	711 (99.0)	541 (99.4)	1682 (98.7)	2150 (99.4)	144 (100.0)	0.026
	1	0 (0.0)	7 (1.0)	3 (0.6)	23 (1.3)	14 (0.6)	0 (0.0)	
Concomitant use of antidepressants (%)	0	337 (67.9)	436 (60.7)	433 (79.6)	1173 (68.8)	1715 (79.3)	102 (70.8)	<0.001
	1	159 (32.1)	282 (39.3)	111 (20.4)	532 (31.2)	449 (20.7)	42 (29.2)	

**Table 2:** Average number of months of follow up for each benzodiazepine treatment.

Benzodiazepine	Average Number of Months of Follow Up
Alprazolam	7.61
Clonazepam	9.09
Diazepam	5.52
Lorazepam	5.99
Midazolam	4.23
Temazepam	6.91

**Table 3:** Head-to-head benzodiazepine comparisons (adjusted with PTSD drugs) using truncating weights. Numbers represented in brackets denote the 99% confidence intervals. Within drug pairs: the first drug is denoted as 1 and the second drug in the pair is represented as 0. A positive estimate correlates to the first drug increasing SRBs in the drug pair.

Drug Pair	Hazard Ratio (Not Adjusted)	*p* Value	FDR-Adjusted *p* Value	Hazard Ratio (Antidepressants Adjusted)	*p* Value	FDR-Adjusted *p* Value
Alprazolam vs. Clonazepam	0.262 (0.092, 0.747)	0.0122	0.0610	0.187 (0.039, 0.890)	0.0351	0.1399
Alprazolam vs. Diazepam	0.300 (0.094, 0.956)	0.0417	0.1251	0.386 (0.129, 1.158)	0.0895	0.2685
Alprazolam vs. Lorazepam	0.344 (0.133, 0.890)	0.0279	0.1046	0.366 (0.142, 0.943)	0.0373	0.1399
Alprazolam vs. Midazolam	0.987 (0.359, 2.716)	0.9802	0.9802	0.736 (0.255, 2.125)	0.5719	0.6599
Alprazolam vs. Temazepam	0.778 (0.371, 1.634)	0.5074	0.7748	0.895 (0.447, 1.793)	0.7549	0.8088
Clonazepam vs. Diazepam	0.979 (0.471, 2.034)	0.9548	0.9802	1.249 (0.579, 2.691)	0.5712	0.6599
Clonazepam vs. Lorazepam	0.991 (0.618, 1.590)	0.9715	0.9802	1.287 (0.628, 2.638)	0.4914	0.6599
Clonazepam vs. Midazolam	2.776 (1.480, 5.212)	0.0015	0.0113	2.373 (1.089, 5.165)	0.0297	0.1399
Clonazepam vs. Temazepam	1.608 (0.564, 4.586)	0.3736	0.7748	1.161 (0.329, 4.100)	0.8163	0.8163
Diazepam vs. Lorazepam	1.067 (0.393, 2.895)	0.8978	0.9802	0.584 (0.277, 1.232)	0.1583	0.3392
Diazepam vs. Midazolam	1.335 (0.626, 2.843)	0.4543	0.7748	1.996 (0.845, 4.716)	0.1152	0.2880
Diazepam vs. Temazepam	2.113 (0.960, 4.651)	0.0631	0.1576	1.680 (0.748, 3.777)	0.2085	0.3909
Lorazepam vs. Midazolam	3.274 (1.824, 5.877)	<0.0001	0.0015	2.670 (1.430, 4.988)	0.0021	0.0315
Lorazepam vs. Temazepam	1.203 (0.438, 3.304)	0.7197	0.9802	1.428 (0.539, 3.777)	0.4738	0.6599
Midazolam vs. Temazepam	0.739 (0.297, 1.842)	0.5165	0.7748	1.399 (0.526, 3.721)	0.5009	0.6599

## Appendix A. Diagnosis Codes

PTSD:

309.81, F43.10, F43.11, F43.12

Suicide-related events/behaviors:

V62.84,R45.851,E950.3,E956,E950.4,E950.0,E958.8,T14.91,E950.9,E958.9,T14.91XA,E950.5,E950.2,E953.0,E958.1,E953.8,E950.1,E950.7,E952.0,E958.0,E957.1,E957.0,E958.5,E952.1,E955.4,T14.91XD,E950.6,E953.9,E955.0,E957.9,E958.7,E958.3,E954,T14.91XS,E951.0,E951.8,E952.8,E953.1,E955.1,E958.6,E958.2,E955.9,E955.2,X83.8XXA,T42.4X2A,T43.592A,T39.1X2A,X78.8XXA,X78.9XXD,T42.6X2A,X78.9XXA,T43.222A,T50.902A,X83.8XXD,T39.312A,T43.212A,T45.0X2A,X78.1XXA,X78.8XXD,T50.992A,T40.2X2A,T43.292A,T43.012A,T39.012A,T42.8X2A,X78.0XXA,T51.0X2A,T40.5X2A,T40.4X2A,T40.1X2A,T44.7X2A,T38.3X2A,T44.6X2A,T48.1X2A,T46.5X2A,T71.162A,T48.3X2A,T43.022A,T44.3X2A,T50.902D,X79.XXXA,T65.92XA,X78.1XXD,T51.92XA,T42.1X2A,T65.892A,T56.892A,T43.622A,X80.XXXA,T42.4X2D,X78.0XXD,T42.72XA,T43.3X2A,X76.XXXD,T48.4X2A,T51.2X2A,T46.4X2A,T39.1X2D,T40.7X2A,T54.92XA,T40.602A,T45.512A,X76.XXXA,T43.222D,T39.392A,T47.1X2A,T50.902S,X74.9XXD,T39.092A,T38.1X2A,X74.9XXA,T39.312D,T38.892A,T43.612A,X82.8XXA,T42.6X2D,T43.632A,T46.1X2A,T45.0X2D,T50.992D,T54.2X2A,T40.3X2A,T39.012D,T43.4X2A,T58.02XA,T43.592D,X81.0XXA,T43.202A,T43.8X2A,T44.992A,T45.2X2A,T40.1X2D,T41.292A,T50.2X2A,T48.6X2A,T50.7X2A,T49.0X2A,T46.3X2A,T42.0X2A,T36.1X2A,T36.0X2A,X74.9XXS,X72.XXXD,T43.012D,T51.8X2A,T51.0X2D,T54.92XS,T54.3X2A,T65.892D,T65.92XD,T65.222D,T50.3X2A,T48.5X2A,T47.0X2A,T46.6X2A,T65.92XS,T54.1X2A,T52.4X2A,T52.0X2A,T55.1X2A,T59.892A,T42.4X2S,T42.3X2A,T36.3X2A,T37.8X2A,T38.2X2A,T38.3X2D,T40.992A,T40.8X2A,T44.4X2A,T43.692A,T45.2X2D,T44.7X2D,T43.502A,T71.192A,X79.XXXD,X83.2XXA,X83.8XXS,X72.XXXA,X71.9XXA,T48.202A,T40.2X2D,X74.8XXS,T48.3X2D,T39.8X2A,T47.4X2A,T47.6X2A,T50.6X2A,T49.6X2D,T43.3X2D,T50.5X2A,X74.01XA,X73.0XXA,T49.6X2A,X72.XXXS,X78.9XXS,T39.92XA,X80.XXXD,X81.8XXA,T39.4X2A,X77.8XXA,T50.2X2D,T43.622D,T43.292D,T45.4X2A,T46.0X2A,T41.3X2A,T42.5X2A,T42.6X2,T46.7X2A,T46.8X2A,T46.5X2D,T43.1X2A,T43.92XA,T40.5X2D,X71.0XXS,X71.3XXA,X71.8XXA,T43.212D,T46.2X2A,T40.8X2D,T40.602D,T43.022D,T44.1X2A,T46.4X2D,T65.222S,T62.0X2A,T71.162D,T51.1X2A,T51.2X2D,T51.2X2S,T52.8X2A,T51.92XD,T50.8X2A,T56.892D,T58.92XA,T54.3X2S,T54.3X2D,T54.0X2A,T55.0X2A,T36.0X2D,T36.4X2A,T38.5X2A,T36.8X2A,T37.5X2A.

## Appendix B. Categories of Comorbid Diseases

Category 1 (ICD9: 291* or 292* or 303* or 304* or (305* and not 305.1))

Category 2 (ICD9: 295* or 301.2)

Category 3 (ICD9: 296* or 298.0 or 300.4 or 301.1 or 309* or 311*)

Category 4 (ICD9: 297* or (298* and not 298.0))

Category 5 (ICD9: 308* or (300* and not 300.4))

Category 6 (ICD9: 301* not 301.1 and not 301.2)

Category 7 (ICD9: 302*)

Category 8 (ICD9: 306* or 316*)

Category 9 (ICD9: 307*)

Category 10 (ICD9: 290* or 293* or 294* or 310*)

Category 11 (ICD9: 299* or 312* or 313* or 314* or 315*)

Category 12 (ICD9: 317* or 318* or 319*)

**Table A1:** Categories of Comorbid Diseases [25].

ICD9 Code	Disease Name	Category	ICD9 Code	Disease Name	Category
291	Alcohol-induced mental disorders	1	301 (not 301.1 or 301.2)	Personality disorders (not Affective personality disorder or Schizoid personality disorder)	6
292	Drug-induced mental disorders	1	302	Sexual and gender identity disorders	7
303	Alcohol dependence syndrome	1	306	Physiological malfunction arising from mental factors	8
304	Drug dependence	1	316	Psychic factor w oth dis.	8
305 (not 305.1)	Nondependent abuse of drugs (not Tobacco use disorder)	1	307	Special symptoms or syndromes not elsewhere classified	9
295	Schizophrenic disorders	2	290	Dementias	10
301.2	Schizoid personality disorder	2	293	Transient mental disorders due to conditions classified elsewhere	10
296	Episodic mood disorders	3	294	Persistent mental disorders due to conditions classified elsewhere	10
298	Depressive type psychosis	3	310	Specific nonpsychotic mental disorders due to brain damage	10
300.4	Dysthymic disorder	3	299	Autistic disorder-current	11
301.1	Affective personality disorder	3	312	Disturbance of conduct not elsewhere classified	11
309	Adjustment reaction	3	313	Disturbance of emotions specific to childhood and adolescence	11
311	Depressive disorder NEC	3	314	Hyperkinetic syndrome of childhood	11
297	Delusional disorders	4	315	Specific delays in development	11
298 (but not 2980)	Other nonorganic psychoses (not Depressive type psychosis)	4	317	Mild intellectual disabilities	12
308	Acute reaction to stress	5	318	Other specified intellectual disabilities	12
300 (but not 300.4)	Anxiety, dissociative and somatoform disorders (not Dysthymic disorder)	5	319	Unspecified intellectual disabilities	12

## Appendix C. Concomitant Antidepressant Therapy

Fluoxetine
Paroxetine
Sertraline
Venlafaxine
Bupropion
Duloxetine
Citalopram
Desvenlafaxine
Duloxetine
Escitalopram
Fluvoxamine
Levomilnacipran
Maprotiline
Milnacipran
Mirtazapine
Nefazodone
Trazodone
Vilazodone
Vortioxetine
Isocarboxazid
Phenelzine
Selegiline
Tranylcypromine
Amitriptyline
Amoxapine
Clomipramine
Desipramine
Doxepin
Imipramine
Nortriptyline
Protriptyline
Trimipramine

## Appendix D

**Table A2:** Effects of Concomitant Medications on the Comparison of Lorazepam and Midazolam by Adjusting 39 Other Drugs.

Name	Estimate	std	lb	ub	z	*p* Value
Intercept	−5.9419	0.54543	−7.011	−4.8729	−10.894	<0.0001
Treatment	0.94076	0.40735	0.14234	1.73917	2.3094	0.0209
Follow-up time	−0.0926	0.02892	−0.1493	−0.0359	−3.202	0.0014
Follow-up time_2	0.00096	0.00023	0.0005	0.00141	4.124	<0.0001
Cat1_b	1.00348	0.24653	0.52027	1.48668	4.0704	<0.0001
Cat2_b	−0.0714	0.40214	−0.8596	0.71676	−0.1776	0.859
Cat4_b	0.34041	0.43494	−0.5121	1.19289	0.7827	0.4338
Cat5_b	0.21344	0.21254	−0.2031	0.63001	1.0042	0.3153
Cat6_b	1.19984	0.33377	0.54565	1.85403	3.5948	0.0003
Cat7_b	0.30272	0.83573	−1.3353	1.94076	0.3622	0.7172
Cat8_b	0.72681	0.75091	−0.745	2.19858	0.9679	0.3331
Cat9_b	1.13729	0.35499	0.44151	1.83307	3.2037	0.0014
Cat10_b	−1.0082	0.36826	−1.7299	−0.2864	−2.7376	0.0062
Cat11_b	−0.1977	0.35469	−0.8929	0.49746	−0.5575	0.5772
Cat12_b	−0.5729	0.49754	−1.5481	0.40228	−1.1515	0.2495
Age_b	−0.0408	0.00778	−0.0561	−0.0256	−5.2439	<0.0001
ED_Visits_3Month_b	0.03936	0.05167	−0.0619	0.14063	0.7618	0.4462
Gender	0.67396	0.22896	0.2252	1.12272	2.9436	0.0032
Acetaminophen_b	0.18996	0.25015	−0.3003	0.68026	0.7594	0.4476
Aripiprazole_b	0.74683	0.33604	0.08819	1.40546	2.2224	0.0263
Aspirin_b	0.0224	0.3614	−0.686	0.73075	0.062	0.9506
Bupropion_b	−0.4841	0.42867	−1.3243	0.35604	−1.1294	0.2587
Citalopram_b	0.72898	0.34973	0.0435	1.41446	2.0844	0.0371
Diphenhydramine_b	−0.0772	0.29636	−0.658	0.50371	−0.2604	0.7946
Escitalopram_b	−0.1451	0.46326	−1.053	0.76293	−0.3131	0.7542
Fentanyl_b	0.47522	0.44267	−0.3924	1.34286	1.0735	0.283
Fluoxetine_b	0.22355	0.33189	−0.427	0.87405	0.6736	0.5006
Gabapentin_b	0.59239	0.23975	0.12248	1.06231	2.4709	0.0135
Haloperidol_b	1.53651	0.24372	1.05882	2.0142	6.3045	<0.0001
Hydrocodone_b	0.30606	0.27333	−0.2297	0.84178	1.1197	0.2628
Hydromorphone_b	0.13635	0.30207	−0.4557	0.72841	0.4514	0.6517
Ibuprofen_b	−0.3626	0.23833	−0.8298	0.1045	−1.5215	0.1281
Lamotrigine_b	0.02815	0.40435	−0.7644	0.82066	0.0696	0.9445
Morphine_b	−0.4032	0.38854	−1.1647	0.35839	−1.0376	0.2995
Naproxen_b	0.25857	0.30863	−0.3464	0.86349	0.8378	0.4021
Olanzapine_b	0.19939	0.38455	−0.5543	0.95311	0.5185	0.6041
Omeprazole_b	0.06562	0.3489	−0.6182	0.74947	0.1881	0.8508
Oxycodone_b	0.4907	0.31773	−0.1321	1.11345	1.5444	0.1225
Quetiapine_b	0.24618	0.3451	−0.4302	0.92256	0.7134	0.4756
Sertraline_b	0.47201	0.29304	−0.1024	1.04637	1.6107	0.1072
Thiamine_b	−1.3291	0.48247	−2.2747	−0.3835	−2.7548	0.0059
Topiramate_b	0.13375	0.42914	−0.7074	0.97486	0.3117	0.7553
Tramadol_b	0.50358	0.3418	−0.1664	1.17351	1.4733	0.1407
Trazodone_b	0.03562	0.24776	−0.45	0.52122	0.1438	0.8857
Venlafaxine_b	1.08102	0.33955	0.41549	1.74654	3.1836	0.0015
Zolpidem_b	0.10398	0.38867	−0.6578	0.86578	0.2675	0.7891
Prazosin_b	−0.1437	0.43278	−0.9919	0.70458	−0.332	0.7399
Choline_b	−0.9793	0.3847	−1.7333	−0.2253	−2.5457	0.0109
Dexmedetomidine_b	−0.5316	0.36263	−1.2424	0.17915	−1.466	0.1427
Meperidine_b	0.65359	0.50608	−0.3383	1.6455	1.2915	0.1965
Metoclopramide_b	−0.6538	0.44609	−1.5281	0.22054	−1.4656	0.1428
Nalbuphine_b	−0.8213	0.35742	−1.5218	−0.1208	−2.2978	0.0216
Naloxone_b	0.30207	0.39818	−0.4784	1.08249	0.7586	0.4481
Perphenazine_b	−0.1299	0.32893	−0.7746	0.51479	−0.395	0.6929
Phenylephrine_b	0.21676	0.35138	−0.472	0.90547	0.6169	0.5373
Ropivacaine_b	0.29554	0.39196	−0.4727	1.06378	0.754	0.4509
Scopolamine_b	−0.5001	0.32562	−1.1384	0.13807	−1.536	0.1245

Drugname_b is a variable indicating whether a patient ever took this drug at baseline. The variable ‘treatment’ indicates treating by lorazepam(1) or midazolam(0). CatN_b (where N = 1 to 12) is the indicator of whether a patient had a diagnosis of disease category N at the baseline time.

## Appendix E

**Table A3:** Information on Concomitant Medications at Baseline.

	Level	Alprazolam	Chlordiazepoxide	Clonazepam	Diazepam	Lorazepam	Midazolam	Temazepam	Triazolam	*p*
n		495	65	717	544	1701	2153	143	51	
Acetaminophen_b (%)	0	337 (68.1)	47 (72.3)	509 (71.0)	340 (62.5)	976 (57.4)	743 (34.5)	86 (60.1)	27 (52.9)	<0.001
	1	158 (31.9)	18 (27.7)	208 (29.0)	204 (37.5)	725 (42.6)	1410 (65.5)	57 (39.9)	24 (47.1)	
Almotriptan_b (%)	0	495 (100.0)	65 (100.0)	717 (100.0)	544 (100.0)	1700 (99.9)	2153 (100.0)	143 (100.0)	51 (100.0)	0.931
	1	0 (0.0)	0 (0.0)	0 (0.0)	0 (0.0)	1 (0.1)	0 (0.0)	0 (0.0)	0 (0.0)	
Alprazolam_b (%)	0	0 (0.0)	65 (100.0)	713 (99.4)	543 (99.8)	1698 (99.8)	2151 (99.9)	143 (100.0)	51 (100.0)	<0.001
	1	495 (100.0)	0 (0.0)	4 (0.6)	1 (0.2)	3 (0.2)	2 (0.1)	0 (0.0)	0 (0.0)	
Amitriptyline_b (%)	0	477 (96.4)	63 (96.9)	684 (95.4)	524 (96.3)	1645 (96.7)	2096 (97.4)	141 (98.6)	50 (98.0)	0.245
	1	18 (3.6)	2 (3.1)	33 (4.6)	20 (3.7)	56 (3.3)	57 (2.6)	2 (1.4)	1 (2.0)	
Amphetamine_b (%)	0	476 (96.2)	64 (98.5)	687 (95.8)	530 (97.4)	1666 (97.9)	2121 (98.5)	139 (97.2)	49 (96.1)	0.001
	1	19 (3.8)	1 (1.5)	30 (4.2)	14 (2.6)	35 (2.1)	32 (1.5)	4 (2.8)	2 (3.9)	
Aripiprazole_b (%)	0	482 (97.4)	61 (93.8)	670 (93.4)	533 (98.0)	1608 (94.5)	2091 (97.1)	134 (93.7)	48 (94.1)	<0.001
	1	13 (2.6)	4 (6.2)	47 (6.6)	11 (2.0)	93 (5.5)	62 (2.9)	9 (6.3)	3 (5.9)	
Armodafinil_b (%)	0	495 (100.0)	65 (100.0)	716 (99.9)	544 (100.0)	1699 (99.9)	2152 (100.0)	143 (100.0)	51 (100.0)	0.949
	1	0 (0.0)	0 (0.0)	1 (0.1)	0 (0.0)	2 (0.1)	1 (0.0)	0 (0.0)	0 (0.0)	
Asenapine_b (%)	0	495 (100.0)	65 (100.0)	715 (99.7)	544 (100.0)	1700 (99.9)	2153 (100.0)	143 (100.0)	51 (100.0)	0.248
	1	0 (0.0)	0 (0.0)	2 (0.3)	0 (0.0)	1 (0.1)	0 (0.0)	0 (0.0)	0 (0.0)	
Aspirin_b (%)	0	452 (91.3)	59 (90.8)	659 (91.9)	511 (93.9)	1475 (86.7)	1887 (87.6)	118 (82.5)	50 (98.0)	<0.001
	1	43 (8.7)	6 (9.2)	58 (8.1)	33 (6.1)	226 (13.3)	266 (12.4)	25 (17.5)	1 (2.0)	
Atomoxetine_b (%)	0	492 (99.4)	64 (98.5)	707 (98.6)	538 (98.9)	1691 (99.4)	2145 (99.6)	141 (98.6)	50 (98.0)	0.077
	1	3 (0.6)	1 (1.5)	10 (1.4)	6 (1.1)	10 (0.6)	8 (0.4)	2 (1.4)	1 (2.0)	
Atropine_b (%)	0	491 (99.2)	54 (83.1)	706 (98.5)	540 (99.3)	1671 (98.2)	2117 (98.3)	133 (93.0)	51 (100.0)	<0.001
	1	4 (0.8)	11 (16.9)	11 (1.5)	4 (0.7)	30 (1.8)	36 (1.7)	10 (7.0)	0 (0.0)	
Benzoate_b (%)	0	488 (98.6)	64 (98.5)	700 (97.6)	538 (98.9)	1691 (99.4)	2142 (99.5)	140 (97.9)	51 (100.0)	<0.001
	1	7 (1.4)	1 (1.5)	17 (2.4)	6 (1.1)	10 (0.6)	11 (0.5)	3 (2.1)	0 (0.0)	
Brexpiprazole_b (%)	0	494 (99.8)	65 (100.0)	715 (99.7)	543 (99.8)	1699 (99.9)	2153 (100.0)	143 (100.0)	51 (100.0)	0.595
	1	1 (0.2)	0 (0.0)	2 (0.3)	1 (0.2)	2 (0.1)	0 (0.0)	0 (0.0)	0 (0.0)	
Brompheniramine_b (%)	0	495 (100.0)	65 (100.0)	715 (99.7)	544 (100.0)	1701 (100.0)	2153 (100.0)	143 (100.0)	51 (100.0)	0.045
	1	0 (0.0)	0 (0.0)	2 (0.3)	0 (0.0)	0 (0.0)	0 (0.0)	0 (0.0)	0 (0.0)	
Buprenorphine_b (%)	0	486 (98.2)	62 (95.4)	700 (97.6)	533 (98.0)	1677 (98.6)	2114 (98.2)	140 (97.9)	50 (98.0)	0.559
	1	9 (1.8)	3 (4.6)	17 (2.4)	11 (2.0)	24 (1.4)	39 (1.8)	3 (2.1)	1 (2.0)	
Bupropion_b (%)	0	450 (90.9)	60 (92.3)	621 (86.6)	498 (91.5)	1576 (92.7)	2015 (93.6)	123 (86.0)	46 (90.2)	<0.001
	1	45 (9.1)	5 (7.7)	96 (13.4)	46 (8.5)	125 (7.3)	138 (6.4)	20 (14.0)	5 (9.8)	
Buspirone_b (%)	0	469 (94.7)	63 (96.9)	671 (93.6)	514 (94.5)	1622 (95.4)	2081 (96.7)	132 (92.3)	46 (90.2)	0.004
	1	26 (5.3)	2 (3.1)	46 (6.4)	30 (5.5)	79 (4.6)	72 (3.3)	11 (7.7)	5 (9.8)	
Butalbital_b (%)	0	493 (99.6)	65 (100.0)	715 (99.7)	543 (99.8)	1701 (100.0)	2153 (100.0)	143 (100.0)	51 (100.0)	0.067
	1	2 (0.4)	0 (0.0)	2 (0.3)	1 (0.2)	0 (0.0)	0 (0.0)	0 (0.0)	0 (0.0)	
Butorphanol_b (%)	0	495 (100.0)	65 (100.0)	715 (99.7)	544 (100.0)	1699 (99.9)	2128 (98.8)	143 (100.0)	51 (100.0)	<0.001
	1	0 (0.0)	0 (0.0)	2 (0.3)	0 (0.0)	2 (0.1)	25 (1.2)	0 (0.0)	0 (0.0)	
Caffeine_b (%)	0	487 (98.4)	65 (100.0)	705 (98.3)	540 (99.3)	1681 (98.8)	2135 (99.2)	142 (99.3)	51 (100.0)	0.414
	1	8 (1.6)	0 (0.0)	12 (1.7)	4 (0.7)	20 (1.2)	18 (0.8)	1 (0.7)	0 (0.0)	
Calcium Carbonate_b (%)	0	487 (98.4)	65 (100.0)	698 (97.4)	533 (98.0)	1676 (98.5)	2102 (97.6)	142 (99.3)	51 (100.0)	0.213
	1	8 (1.6)	0 (0.0)	19 (2.6)	11 (2.0)	25 (1.5)	51 (2.4)	1 (0.7)	0 (0.0)	
Carbamazepine_b (%)	0	488 (98.6)	65 (100.0)	710 (99.0)	537 (98.7)	1678 (98.6)	2139 (99.3)	143 (100.0)	51 (100.0)	0.266
	1	7 (1.4)	0 (0.0)	7 (1.0)	7 (1.3)	23 (1.4)	14 (0.7)	0 (0.0)	0 (0.0)	
Carbidopa_b (%)	0	495 (100.0)	65 (100.0)	715 (99.7)	543 (99.8)	1700 (99.9)	2151 (99.9)	142 (99.3)	51 (100.0)	0.414
	1	0 (0.0)	0 (0.0)	2 (0.3)	1 (0.2)	1 (0.1)	2 (0.1)	1 (0.7)	0 (0.0)	
Cariprazine_b (%)	0	495 (100.0)	65 (100.0)	717 (100.0)	543 (99.8)	1700 (99.9)	2152 (100.0)	143 (100.0)	51 (100.0)	0.915
	1	0 (0.0)	0 (0.0)	0 (0.0)	1 (0.2)	1 (0.1)	1 (0.0)	0 (0.0)	0 (0.0)	
Carisoprodol_b (%)	0	488 (98.6)	65 (100.0)	713 (99.4)	535 (98.3)	1688 (99.2)	2142 (99.5)	143 (100.0)	51 (100.0)	0.075
	1	7 (1.4)	0 (0.0)	4 (0.6)	9 (1.7)	13 (0.8)	11 (0.5)	0 (0.0)	0 (0.0)	
Chlordiazepoxide_b (%)	0	495 (100.0)	0 (0.0)	717 (100.0)	543 (99.8)	1701 (100.0)	2153 (100.0)	143 (100.0)	51 (100.0)	<0.001
	1	0 (0.0)	65 (100.0)	0 (0.0)	1 (0.2)	0 (0.0)	0 (0.0)	0 (0.0)	0 (0.0)	
Chlorpheniramine_b (%)	0	495 (100.0)	65 (100.0)	714 (99.6)	544 (100.0)	1699 (99.9)	2153 (100.0)	143 (100.0)	51 (100.0)	0.085
	1	0 (0.0)	0 (0.0)	3 (0.4)	0 (0.0)	2 (0.1)	0 (0.0)	0 (0.0)	0 (0.0)	
Chlorpromazine_b (%)	0	494 (99.8)	65 (100.0)	710 (99.0)	541 (99.4)	1689 (99.3)	2149 (99.8)	141 (98.6)	51 (100.0)	0.073
	1	1 (0.2)	0 (0.0)	7 (1.0)	3 (0.6)	12 (0.7)	4 (0.2)	2 (1.4)	0 (0.0)	
Chlorthalidone_b (%)	0	493 (99.6)	64 (98.5)	712 (99.3)	543 (99.8)	1695 (99.6)	2149 (99.8)	143 (100.0)	51 (100.0)	0.295
	1	2 (0.4)	1 (1.5)	5 (0.7)	1 (0.2)	6 (0.4)	4 (0.2)	0 (0.0)	0 (0.0)	
Choline_b (%)	0	493 (99.6)	65 (100.0)	715 (99.7)	539 (99.1)	1682 (98.9)	1572 (73.0)	142 (99.3)	50 (98.0)	<0.001
	1	2 (0.4)	0 (0.0)	2 (0.3)	5 (0.9)	19 (1.1)	581 (27.0)	1 (0.7)	1 (2.0)	
Citalopram_b (%)	0	404 (81.6)	58 (89.2)	591 (82.4)	499 (91.7)	1462 (85.9)	1954 (90.8)	123 (86.0)	46 (90.2)	<0.001
	1	91 (18.4)	7 (10.8)	126 (17.6)	45 (8.3)	239 (14.1)	199 (9.2)	20 (14.0)	5 (9.8)	
Clidinium_b (%)	0	494 (99.8)	62 (95.4)	717 (100.0)	542 (99.6)	1697 (99.8)	2148 (99.8)	143 (100.0)	51 (100.0)	<0.001
	1	1 (0.2)	3 (4.6)	0 (0.0)	2 (0.4)	4 (0.2)	5 (0.2)	0 (0.0)	0 (0.0)	
Clobazam_b (%)	0	495 (100.0)	65 (100.0)	716 (99.9)	544 (100.0)	1701 (100.0)	2153 (100.0)	143 (100.0)	51 (100.0)	0.41
	1	0 (0.0)	0 (0.0)	1 (0.1)	0 (0.0)	0 (0.0)	0 (0.0)	0 (0.0)	0 (0.0)	
Clomipramine_b (%)	0	495 (100.0)	64 (98.5)	714 (99.6)	544 (100.0)	1698 (99.8)	2152 (100.0)	143 (100.0)	51 (100.0)	0.019
	1	0 (0.0)	1 (1.5)	3 (0.4)	0 (0.0)	3 (0.2)	1 (0.0)	0 (0.0)	0 (0.0)	
Clonazepam_b (%)	0	495 (100.0)	65 (100.0)	0 (0.0)	542 (99.6)	1697 (99.8)	2151 (99.9)	142 (99.3)	50 (98.0)	<0.001
	1	0 (0.0)	0 (0.0)	717 (100.0)	2 (0.4)	4 (0.2)	2 (0.1)	1 (0.7)	1 (2.0)	
Clonidine_b (%)	0	486 (98.2)	43 (66.2)	694 (96.8)	527 (96.9)	1626 (95.6)	2103 (97.7)	117 (81.8)	48 (94.1)	<0.001
	1	9 (1.8)	22 (33.8)	23 (3.2)	17 (3.1)	75 (4.4)	50 (2.3)	26 (18.2)	3 (5.9)	
Clozapine_b (%)	0	495 (100.0)	65 (100.0)	715 (99.7)	544 (100.0)	1694 (99.6)	2144 (99.6)	142 (99.3)	51 (100.0)	0.609
	1	0 (0.0)	0 (0.0)	2 (0.3)	0 (0.0)	7 (0.4)	9 (0.4)	1 (0.7)	0 (0.0)	
Codeine_b (%)	0	482 (97.4)	64 (98.5)	703 (98.0)	532 (97.8)	1674 (98.4)	2087 (96.9)	138 (96.5)	49 (96.1)	0.131
	1	13 (2.6)	1 (1.5)	14 (2.0)	12 (2.2)	27 (1.6)	66 (3.1)	5 (3.5)	2 (3.9)	
Desipramine_b (%)	0	495 (100.0)	65 (100.0)	715 (99.7)	544 (100.0)	1699 (99.9)	2149 (99.8)	143 (100.0)	51 (100.0)	0.859
	1	0 (0.0)	0 (0.0)	2 (0.3)	0 (0.0)	2 (0.1)	4 (0.2)	0 (0.0)	0 (0.0)	
Desvenlafaxine_b (%)	0	491 (99.2)	65 (100.0)	714 (99.6)	542 (99.6)	1692 (99.5)	2148 (99.8)	142 (99.3)	51 (100.0)	0.662
	1	4 (0.8)	0 (0.0)	3 (0.4)	2 (0.4)	9 (0.5)	5 (0.2)	1 (0.7)	0 (0.0)	
Dexmedetomidine_b (%)	0	494 (99.8)	65 (100.0)	717 (100.0)	541 (99.4)	1690 (99.4)	1809 (84.0)	143 (100.0)	51 (100.0)	<0.001
	1	1 (0.2)	0 (0.0)	0 (0.0)	3 (0.6)	11 (0.6)	344 (16.0)	0 (0.0)	0 (0.0)	
Dexmethylphenidate_b (%)	0	495 (100.0)	65 (100.0)	717 (100.0)	544 (100.0)	1700 (99.9)	2151 (99.9)	143 (100.0)	51 (100.0)	0.971
	1	0 (0.0)	0 (0.0)	0 (0.0)	0 (0.0)	1 (0.1)	2 (0.1)	0 (0.0)	0 (0.0)	
Dextroamphetamine_b (%)	0	476 (96.2)	64 (98.5)	687 (95.8)	530 (97.4)	1666 (97.9)	2122 (98.6)	139 (97.2)	49 (96.1)	0.001
	1	19 (3.8)	1 (1.5)	30 (4.2)	14 (2.6)	35 (2.1)	31 (1.4)	4 (2.8)	2 (3.9)	
Dextromethorphan_b (%)	0	492 (99.4)	65 (100.0)	711 (99.2)	541 (99.4)	1692 (99.5)	2144 (99.6)	143 (100.0)	51 (100.0)	0.855
	1	3 (0.6)	0 (0.0)	6 (0.8)	3 (0.6)	9 (0.5)	9 (0.4)	0 (0.0)	0 (0.0)	
Diazepam_b (%)	0	495 (100.0)	64 (98.5)	717 (100.0)	0 (0.0)	1701 (100.0)	2152 (100.0)	143 (100.0)	51 (100.0)	<0.001
	1	0 (0.0)	1 (1.5)	0 (0.0)	544 (100.0)	0 (0.0)	1 (0.0)	0 (0.0)	0 (0.0)	
Dihydroergotamine_b (%)	0	495 (100.0)	65 (100.0)	717 (100.0)	543 (99.8)	1698 (99.8)	2152 (100.0)	143 (100.0)	51 (100.0)	0.789
	1	0 (0.0)	0 (0.0)	0 (0.0)	1 (0.2)	3 (0.2)	1 (0.0)	0 (0.0)	0 (0.0)	
Dimenhydrinate_b (%)	0	495 (100.0)	65 (100.0)	716 (99.9)	544 (100.0)	1701 (100.0)	2127 (98.8)	143 (100.0)	51 (100.0)	<0.001
	1	0 (0.0)	0 (0.0)	1 (0.1)	0 (0.0)	0 (0.0)	26 (1.2)	0 (0.0)	0 (0.0)	
Diphenhydramine_b (%)	0	479 (96.8)	62 (95.4)	674 (94.0)	507 (93.2)	1511 (88.8)	1606 (74.6)	133 (93.0)	49 (96.1)	<0.001
	1	16 (3.2)	3 (4.6)	43 (6.0)	37 (6.8)	190 (11.2)	547 (25.4)	10 (7.0)	2 (3.9)	
Dipyridamole_b (%)	0	495 (100.0)	65 (100.0)	717 (100.0)	544 (100.0)	1700 (99.9)	2150 (99.9)	142 (99.3)	51 (100.0)	0.266
	1	0 (0.0)	0 (0.0)	0 (0.0)	0 (0.0)	1 (0.1)	3 (0.1)	1 (0.7)	0 (0.0)	
Donepezil_b (%)	0	492 (99.4)	65 (100.0)	714 (99.6)	543 (99.8)	1692 (99.5)	2151 (99.9)	143 (100.0)	51 (100.0)	0.269
	1	3 (0.6)	0 (0.0)	3 (0.4)	1 (0.2)	9 (0.5)	2 (0.1)	0 (0.0)	0 (0.0)	
Doxepin_b (%)	0	483 (97.6)	57 (87.7)	701 (97.8)	536 (98.5)	1678 (98.6)	2137 (99.3)	139 (97.2)	51 (100.0)	<0.001
	1	12 (2.4)	8 (12.3)	16 (2.2)	8 (1.5)	23 (1.4)	16 (0.7)	4 (2.8)	0 (0.0)	
Doxylamine_b (%)	0	495 (100.0)	65 (100.0)	717 (100.0)	544 (100.0)	1694 (99.6)	2142 (99.5)	143 (100.0)	51 (100.0)	0.203
	1	0 (0.0)	0 (0.0)	0 (0.0)	0 (0.0)	7 (0.4)	11 (0.5)	0 (0.0)	0 (0.0)	
Duloxetine_b (%)	0	461 (93.1)	61 (93.8)	649 (90.5)	509 (93.6)	1624 (95.5)	2074 (96.3)	135 (94.4)	45 (88.2)	<0.001
	1	34 (6.9)	4 (6.2)	68 (9.5)	35 (6.4)	77 (4.5)	79 (3.7)	8 (5.6)	6 (11.8)	
Eletriptan_b (%)	0	494 (99.8)	65 (100.0)	716 (99.9)	544 (100.0)	1700 (99.9)	2152 (100.0)	141 (98.6)	51 (100.0)	0.001
	1	1 (0.2)	0 (0.0)	1 (0.1)	0 (0.0)	1 (0.1)	1 (0.0)	2 (1.4)	0 (0.0)	
Ergotamine_b (%)	0	495 (100.0)	65 (100.0)	717 (100.0)	543 (99.8)	1698 (99.8)	2152 (100.0)	143 (100.0)	51 (100.0)	0.789
	1	0 (0.0)	0 (0.0)	0 (0.0)	1 (0.2)	3 (0.2)	1 (0.0)	0 (0.0)	0 (0.0)	
Escitalopram_b (%)	0	458 (92.5)	61 (93.8)	664 (92.6)	527 (96.9)	1602 (94.2)	2077 (96.5)	134 (93.7)	47 (92.2)	<0.001
	1	37 (7.5)	4 (6.2)	53 (7.4)	17 (3.1)	99 (5.8)	76 (3.5)	9 (6.3)	4 (7.8)	
Eszopiclone_b (%)	0	493 (99.6)	65 (100.0)	715 (99.7)	540 (99.3)	1695 (99.6)	2152 (100.0)	141 (98.6)	51 (100.0)	0.036
	1	2 (0.4)	0 (0.0)	2 (0.3)	4 (0.7)	6 (0.4)	1 (0.0)	2 (1.4)	0 (0.0)	
Fentanyl_b (%)	0	478 (96.6)	64 (98.5)	692 (96.5)	528 (97.1)	1593 (93.7)	191 (8.9)	137 (95.8)	49 (96.1)	<0.001
	1	17 (3.4)	1 (1.5)	25 (3.5)	16 (2.9)	108 (6.3)	1962 (91.1)	6 (4.2)	2 (3.9)	
Fluoxetine_b (%)	0	452 (91.3)	64 (98.5)	648 (90.4)	518 (95.2)	1562 (91.8)	2025 (94.1)	128 (89.5)	42 (82.4)	<0.001
	1	43 (8.7)	1 (1.5)	69 (9.6)	26 (4.8)	139 (8.2)	128 (5.9)	15 (10.5)	9 (17.6)	
Fluphenazine_b (%)	0	494 (99.8)	65 (100.0)	714 (99.6)	541 (99.4)	1697 (99.8)	2153 (100.0)	143 (100.0)	51 (100.0)	0.155
	1	1 (0.2)	0 (0.0)	3 (0.4)	3 (0.6)	4 (0.2)	0 (0.0)	0 (0.0)	0 (0.0)	
Fluvoxamine_b (%)	0	493 (99.6)	65 (100.0)	709 (98.9)	543 (99.8)	1691 (99.4)	2149 (99.8)	142 (99.3)	51 (100.0)	0.073
	1	2 (0.4)	0 (0.0)	8 (1.1)	1 (0.2)	10 (0.6)	4 (0.2)	1 (0.7)	0 (0.0)	
Frovatriptan_b (%)	0	495 (100.0)	65 (100.0)	717 (100.0)	543 (99.8)	1701 (100.0)	2153 (100.0)	143 (100.0)	51 (100.0)	0.201
	1	0 (0.0)	0 (0.0)	0 (0.0)	1 (0.2)	0 (0.0)	0 (0.0)	0 (0.0)	0 (0.0)	
Gabapentin_b (%)	0	428 (86.5)	50 (76.9)	592 (82.6)	435 (80.0)	1460 (85.8)	1838 (85.4)	111 (77.6)	44 (86.3)	0.001
	1	67 (13.5)	15 (23.1)	125 (17.4)	109 (20.0)	241 (14.2)	315 (14.6)	32 (22.4)	7 (13.7)	
Galantamine_b (%)	0	494 (99.8)	65 (100.0)	717 (100.0)	543 (99.8)	1700 (99.9)	2153 (100.0)	143 (100.0)	51 (100.0)	0.575
	1	1 (0.2)	0 (0.0)	0 (0.0)	1 (0.2)	1 (0.1)	0 (0.0)	0 (0.0)	0 (0.0)	
Glycine_b (%)	0	495 (100.0)	65 (100.0)	717 (100.0)	544 (100.0)	1701 (100.0)	2152 (100.0)	143 (100.0)	51 (100.0)	0.973
	1	0 (0.0)	0 (0.0)	0 (0.0)	0 (0.0)	0 (0.0)	1 (0.0)	0 (0.0)	0 (0.0)	
Guaifenesin_b (%)	0	480 (97.0)	64 (98.5)	696 (97.1)	533 (98.0)	1648 (96.9)	2105 (97.8)	139 (97.2)	49 (96.1)	0.673
	1	15 (3.0)	1 (1.5)	21 (2.9)	11 (2.0)	53 (3.1)	48 (2.2)	4 (2.8)	2 (3.9)	
Haloperidol_b (%)	0	494 (99.8)	63 (96.9)	706 (98.5)	543 (99.8)	1499 (88.1)	2086 (96.9)	139 (97.2)	51 (100.0)	<0.001
	1	1 (0.2)	2 (3.1)	11 (1.5)	1 (0.2)	202 (11.9)	67 (3.1)	4 (2.8)	0 (0.0)	
Homatropine_b (%)	0	492 (99.4)	65 (100.0)	713 (99.4)	542 (99.6)	1692 (99.5)	2146 (99.7)	142 (99.3)	51 (100.0)	0.943
	1	3 (0.6)	0 (0.0)	4 (0.6)	2 (0.4)	9 (0.5)	7 (0.3)	1 (0.7)	0 (0.0)	
Hydrocodone_b (%)	0	421 (85.1)	60 (92.3)	645 (90.0)	448 (82.4)	1530 (89.9)	1790 (83.1)	125 (87.4)	32 (62.7)	<0.001
	1	74 (14.9)	5 (7.7)	72 (10.0)	96 (17.6)	171 (10.1)	363 (16.9)	18 (12.6)	19 (37.3)	
Hydromorphone_b (%)	0	470 (94.9)	63 (96.9)	682 (95.1)	462 (84.9)	1518 (89.2)	1032 (47.9)	129 (90.2)	51 (100.0)	<0.001
	1	25 (5.1)	2 (3.1)	35 (4.9)	82 (15.1)	183 (10.8)	1121 (52.1)	14 (9.8)	0 (0.0)	
Hyoscyamine_b (%)	0	492 (99.4)	64 (98.5)	712 (99.3)	539 (99.1)	1694 (99.6)	2145 (99.6)	143 (100.0)	50 (98.0)	0.359
	1	3 (0.6)	1 (1.5)	5 (0.7)	5 (0.9)	7 (0.4)	8 (0.4)	0 (0.0)	1 (2.0)	
Ibuprofen_b (%)	0	420 (84.8)	48 (73.8)	617 (86.1)	419 (77.0)	1398 (82.2)	1550 (72.0)	108 (75.5)	39 (76.5)	<0.001
	1	75 (15.2)	17 (26.2)	100 (13.9)	125 (23.0)	303 (17.8)	603 (28.0)	35 (24.5)	12 (23.5)	
Iloperidone_b (%)	0	495 (100.0)	65 (100.0)	716 (99.9)	544 (100.0)	1701 (100.0)	2153 (100.0)	143 (100.0)	51 (100.0)	0.41
	1	0 (0.0)	0 (0.0)	1 (0.1)	0 (0.0)	0 (0.0)	0 (0.0)	0 (0.0)	0 (0.0)	
Imipramine_b (%)	0	495 (100.0)	65 (100.0)	713 (99.4)	543 (99.8)	1697 (99.8)	2151 (99.9)	143 (100.0)	51 (100.0)	0.34
	1	0 (0.0)	0 (0.0)	4 (0.6)	1 (0.2)	4 (0.2)	2 (0.1)	0 (0.0)	0 (0.0)	
Lacosamide_b (%)	0	495 (100.0)	65 (100.0)	716 (99.9)	544 (100.0)	1697 (99.8)	2151 (99.9)	143 (100.0)	51 (100.0)	0.823
	1	0 (0.0)	0 (0.0)	1 (0.1)	0 (0.0)	4 (0.2)	2 (0.1)	0 (0.0)	0 (0.0)	
Lamotrigine_b (%)	0	477 (96.4)	62 (95.4)	643 (89.7)	523 (96.1)	1605 (94.4)	2086 (96.9)	135 (94.4)	47 (92.2)	<0.001
	1	18 (3.6)	3 (4.6)	74 (10.3)	21 (3.9)	96 (5.6)	67 (3.1)	8 (5.6)	4 (7.8)	
Levetiracetam_b (%)	0	491 (99.2)	65 (100.0)	705 (98.3)	536 (98.5)	1629 (95.8)	2130 (98.9)	142 (99.3)	51 (100.0)	<0.001
	1	4 (0.8)	0 (0.0)	12 (1.7)	8 (1.5)	72 (4.2)	23 (1.1)	1 (0.7)	0 (0.0)	
Levodopa_b (%)	0	495 (100.0)	65 (100.0)	717 (100.0)	543 (99.8)	1701 (100.0)	2152 (100.0)	142 (99.3)	51 (100.0)	0.034
	1	0 (0.0)	0 (0.0)	0 (0.0)	1 (0.2)	0 (0.0)	1 (0.0)	1 (0.7)	0 (0.0)	
Levomilnacipran_b (%)	0	495 (100.0)	65 (100.0)	717 (100.0)	544 (100.0)	1700 (99.9)	2152 (100.0)	143 (100.0)	51 (100.0)	0.993
	1	0 (0.0)	0 (0.0)	0 (0.0)	0 (0.0)	1 (0.1)	1 (0.0)	0 (0.0)	0 (0.0)	
Lisdexamfetamine_b (%)	0	491 (99.2)	64 (98.5)	711 (99.2)	541 (99.4)	1686 (99.1)	2148 (99.8)	142 (99.3)	51 (100.0)	0.215
	1	4 (0.8)	1 (1.5)	6 (0.8)	3 (0.6)	15 (0.9)	5 (0.2)	1 (0.7)	0 (0.0)	
Lithium_b (%)	0	486 (98.2)	64 (98.5)	682 (95.1)	535 (98.3)	1651 (97.1)	2129 (98.9)	139 (97.2)	51 (100.0)	<0.001
	1	9 (1.8)	1 (1.5)	35 (4.9)	9 (1.7)	50 (2.9)	24 (1.1)	4 (2.8)	0 (0.0)	
Lorazepam_b (%)	0	493 (99.6)	65 (100.0)	716 (99.9)	544 (100.0)	0 (0.0)	2151 (99.9)	142 (99.3)	51 (100.0)	<0.001
	1	2 (0.4)	0 (0.0)	1 (0.1)	0 (0.0)	1701 (100.0)	2 (0.1)	1 (0.7)	0 (0.0)	
Loxapine_b (%)	0	495 (100.0)	65 (100.0)	716 (99.9)	544 (100.0)	1701 (100.0)	2153 (100.0)	143 (100.0)	51 (100.0)	0.41
	1	0 (0.0)	0 (0.0)	1 (0.1)	0 (0.0)	0 (0.0)	0 (0.0)	0 (0.0)	0 (0.0)	
Lurasidone_b (%)	0	490 (99.0)	64 (98.5)	709 (98.9)	535 (98.3)	1683 (98.9)	2144 (99.6)	141 (98.6)	50 (98.0)	0.126
	1	5 (1.0)	1 (1.5)	8 (1.1)	9 (1.7)	18 (1.1)	9 (0.4)	2 (1.4)	1 (2.0)	
Magnesium Carbonate_b (%)	0	495 (100.0)	65 (100.0)	717 (100.0)	543 (99.8)	1701 (100.0)	2152 (100.0)	142 (99.3)	51 (100.0)	0.034
	1	0 (0.0)	0 (0.0)	0 (0.0)	1 (0.2)	0 (0.0)	1 (0.0)	1 (0.7)	0 (0.0)	
Meclizine_b (%)	0	487 (98.4)	65 (100.0)	705 (98.3)	524 (96.3)	1657 (97.4)	2134 (99.1)	142 (99.3)	51 (100.0)	<0.001
	1	8 (1.6)	0 (0.0)	12 (1.7)	20 (3.7)	44 (2.6)	19 (0.9)	1 (0.7)	0 (0.0)	
Melatonin_b (%)	0	484 (97.8)	63 (96.9)	698 (97.4)	531 (97.6)	1625 (95.5)	2098 (97.4)	141 (98.6)	51 (100.0)	0.009
	1	11 (2.2)	2 (3.1)	19 (2.6)	13 (2.4)	76 (4.5)	55 (2.6)	2 (1.4)	0 (0.0)	
Memantine_b (%)	0	494 (99.8)	65 (100.0)	717 (100.0)	544 (100.0)	1700 (99.9)	2152 (100.0)	142 (99.3)	51 (100.0)	0.149
	1	1 (0.2)	0 (0.0)	0 (0.0)	0 (0.0)	1 (0.1)	1 (0.0)	1 (0.7)	0 (0.0)	
Meperidine_b (%)	0	493 (99.6)	65 (100.0)	717 (100.0)	544 (100.0)	1694 (99.6)	1957 (90.9)	142 (99.3)	51 (100.0)	<0.001
	1	2 (0.4)	0 (0.0)	0 (0.0)	0 (0.0)	7 (0.4)	196 (9.1)	1 (0.7)	0 (0.0)	
Methadone_b (%)	0	485 (98.0)	65 (100.0)	700 (97.6)	537 (98.7)	1662 (97.7)	2107 (97.9)	140 (97.9)	51 (100.0)	0.676
	1	10 (2.0)	0 (0.0)	17 (2.4)	7 (1.3)	39 (2.3)	46 (2.1)	3 (2.1)	0 (0.0)	
Methocarbamol_b (%)	0	486 (98.2)	63 (96.9)	704 (98.2)	521 (95.8)	1678 (98.6)	2113 (98.1)	140 (97.9)	51 (100.0)	0.006
	1	9 (1.8)	2 (3.1)	13 (1.8)	23 (4.2)	23 (1.4)	40 (1.9)	3 (2.1)	0 (0.0)	
Methylergonovine_b (%)	0	495 (100.0)	65 (100.0)	715 (99.7)	544 (100.0)	1701 (100.0)	2131 (99.0)	143 (100.0)	51 (100.0)	<0.001
	1	0 (0.0)	0 (0.0)	2 (0.3)	0 (0.0)	0 (0.0)	22 (1.0)	0 (0.0)	0 (0.0)	
Methylnaltrexone_b (%)	0	495 (100.0)	65 (100.0)	717 (100.0)	544 (100.0)	1700 (99.9)	2153 (100.0)	143 (100.0)	51 (100.0)	0.931
	1	0 (0.0)	0 (0.0)	0 (0.0)	0 (0.0)	1 (0.1)	0 (0.0)	0 (0.0)	0 (0.0)	
Methylphenidate_b (%)	0	489 (98.8)	65 (100.0)	698 (97.4)	540 (99.3)	1674 (98.4)	2134 (99.1)	142 (99.3)	47 (92.2)	<0.001
	1	6 (1.2)	0 (0.0)	19 (2.6)	4 (0.7)	27 (1.6)	19 (0.9)	1 (0.7)	4 (7.8)	
Metoclopramide_b (%)	0	485 (98.0)	65 (100.0)	703 (98.0)	528 (97.1)	1635 (96.1)	1991 (92.5)	141 (98.6)	51 (100.0)	<0.001
	1	10 (2.0)	0 (0.0)	14 (2.0)	16 (2.9)	66 (3.9)	162 (7.5)	2 (1.4)	0 (0.0)	
Midazolam_b (%)	0	495 (100.0)	65 (100.0)	717 (100.0)	544 (100.0)	1701 (100.0)	0 (0.0)	142 (99.3)	51 (100.0)	<0.001
	1	0 (0.0)	0 (0.0)	0 (0.0)	0 (0.0)	0 (0.0)	2153 (100.0)	1 (0.7)	0 (0.0)	
Milnacipran_b (%)	0	494 (99.8)	65 (100.0)	716 (99.9)	544 (100.0)	1699 (99.9)	2152 (100.0)	142 (99.3)	51 (100.0)	0.435
	1	1 (0.2)	0 (0.0)	1 (0.1)	0 (0.0)	2 (0.1)	1 (0.0)	1 (0.7)	0 (0.0)	
Mirtazapine_b (%)	0	476 (96.2)	58 (89.2)	672 (93.7)	524 (96.3)	1619 (95.2)	2107 (97.9)	135 (94.4)	50 (98.0)	<0.001
	1	19 (3.8)	7 (10.8)	45 (6.3)	20 (3.7)	82 (4.8)	46 (2.1)	8 (5.6)	1 (2.0)	
Modafinil_b (%)	0	492 (99.4)	65 (100.0)	713 (99.4)	544 (100.0)	1696 (99.7)	2149 (99.8)	143 (100.0)	51 (100.0)	0.43
	1	3 (0.6)	0 (0.0)	4 (0.6)	0 (0.0)	5 (0.3)	4 (0.2)	0 (0.0)	0 (0.0)	
Morphine_b (%)	0	473 (95.6)	65 (100.0)	691 (96.4)	510 (93.8)	1588 (93.4)	1628 (75.6)	132 (92.3)	51 (100.0)	<0.001
	1	22 (4.4)	0 (0.0)	26 (3.6)	34 (6.2)	113 (6.6)	525 (24.4)	11 (7.7)	0 (0.0)	
Nalbuphine_b (%)	0	493 (99.6)	65 (100.0)	714 (99.6)	544 (100.0)	1685 (99.1)	1904 (88.4)	143 (100.0)	51 (100.0)	<0.001
	1	2 (0.4)	0 (0.0)	3 (0.4)	0 (0.0)	16 (0.9)	249 (11.6)	0 (0.0)	0 (0.0)	
Naloxone_b (%)	0	482 (97.4)	61 (93.8)	695 (96.9)	531 (97.6)	1647 (96.8)	1873 (87.0)	140 (97.9)	49 (96.1)	<0.001
	1	13 (2.6)	4 (6.2)	22 (3.1)	13 (2.4)	54 (3.2)	280 (13.0)	3 (2.1)	2 (3.9)	
Naltrexone_b (%)	0	493 (99.6)	59 (90.8)	715 (99.7)	539 (99.1)	1687 (99.2)	2144 (99.6)	143 (100.0)	51 (100.0)	<0.001
	1	2 (0.4)	6 (9.2)	2 (0.3)	5 (0.9)	14 (0.8)	9 (0.4)	0 (0.0)	0 (0.0)	
Naproxen_b (%)	0	469 (94.7)	61 (93.8)	680 (94.8)	493 (90.6)	1596 (93.8)	2037 (94.6)	137 (95.8)	50 (98.0)	0.022
	1	26 (5.3)	4 (6.2)	37 (5.2)	51 (9.4)	105 (6.2)	116 (5.4)	6 (4.2)	1 (2.0)	
Naratriptan_b (%)	0	493 (99.6)	65 (100.0)	716 (99.9)	544 (100.0)	1697 (99.8)	2151 (99.9)	143 (100.0)	51 (100.0)	0.717
	1	2 (0.4)	0 (0.0)	1 (0.1)	0 (0.0)	4 (0.2)	2 (0.1)	0 (0.0)	0 (0.0)	
Nortriptyline_b (%)	0	490 (99.0)	65 (100.0)	703 (98.0)	537 (98.7)	1674 (98.4)	2135 (99.2)	142 (99.3)	50 (98.0)	0.247
	1	5 (1.0)	0 (0.0)	14 (2.0)	7 (1.3)	27 (1.6)	18 (0.8)	1 (0.7)	1 (2.0)	
Olanzapine_b (%)	0	484 (97.8)	65 (100.0)	689 (96.1)	535 (98.3)	1610 (94.7)	2117 (98.3)	142 (99.3)	51 (100.0)	<0.001
	1	11 (2.2)	0 (0.0)	28 (3.9)	9 (1.7)	91 (5.3)	36 (1.7)	1 (0.7)	0 (0.0)	
Omeprazole_b (%)	0	432 (87.3)	58 (89.2)	623 (86.9)	473 (86.9)	1515 (89.1)	1889 (87.7)	122 (85.3)	44 (86.3)	0.701
	1	63 (12.7)	7 (10.8)	94 (13.1)	71 (13.1)	186 (10.9)	264 (12.3)	21 (14.7)	7 (13.7)	
Opium_b (%)	0	476 (96.2)	64 (98.5)	692 (96.5)	529 (97.2)	1648 (96.9)	2093 (97.2)	136 (95.1)	51 (100.0)	0.551
	1	19 (3.8)	1 (1.5)	25 (3.5)	15 (2.8)	53 (3.1)	60 (2.8)	7 (4.9)	0 (0.0)	
Orphenadrine_b (%)	0	492 (99.4)	65 (100.0)	713 (99.4)	536 (98.5)	1694 (99.6)	2148 (99.8)	143 (100.0)	51 (100.0)	0.023
	1	3 (0.6)	0 (0.0)	4 (0.6)	8 (1.5)	7 (0.4)	5 (0.2)	0 (0.0)	0 (0.0)	
Oxcarbazepine_b (%)	0	491 (99.2)	65 (100.0)	710 (99.0)	537 (98.7)	1679 (98.7)	2140 (99.4)	141 (98.6)	51 (100.0)	0.414
	1	4 (0.8)	0 (0.0)	7 (1.0)	7 (1.3)	22 (1.3)	13 (0.6)	2 (1.4)	0 (0.0)	
Oxycodone_b (%)	0	435 (87.9)	58 (89.2)	630 (87.9)	413 (75.9)	1498 (88.1)	1213 (56.3)	127 (88.8)	42 (82.4)	<0.001
	1	60 (12.1)	7 (10.8)	87 (12.1)	131 (24.1)	203 (11.9)	940 (43.7)	16 (11.2)	9 (17.6)	
Oxymorphone_b (%)	0	490 (99.0)	65 (100.0)	717 (100.0)	542 (99.6)	1697 (99.8)	2141 (99.4)	142 (99.3)	51 (100.0)	0.159
	1	5 (1.0)	0 (0.0)	0 (0.0)	2 (0.4)	4 (0.2)	12 (0.6)	1 (0.7)	0 (0.0)	
Paliperidone_b (%)	0	495 (100.0)	65 (100.0)	714 (99.6)	543 (99.8)	1692 (99.5)	2149 (99.8)	140 (97.9)	51 (100.0)	0.008
	1	0 (0.0)	0 (0.0)	3 (0.4)	1 (0.2)	9 (0.5)	4 (0.2)	3 (2.1)	0 (0.0)	
Paroxetine_b (%)	0	472 (95.4)	63 (96.9)	685 (95.5)	531 (97.6)	1638 (96.3)	2106 (97.8)	138 (96.5)	50 (98.0)	0.016
	1	23 (4.6)	2 (3.1)	32 (4.5)	13 (2.4)	63 (3.7)	47 (2.2)	5 (3.5)	1 (2.0)	
Perphenazine_b (%)	0	493 (99.6)	64 (98.5)	714 (99.6)	538 (98.9)	1693 (99.5)	2041 (94.8)	142 (99.3)	51 (100.0)	<0.001
	1	2 (0.4)	1 (1.5)	3 (0.4)	6 (1.1)	8 (0.5)	112 (5.2)	1 (0.7)	0 (0.0)	
Phenazopyridine_b (%)	0	490 (99.0)	65 (100.0)	711 (99.2)	542 (99.6)	1690 (99.4)	2120 (98.5)	143 (100.0)	51 (100.0)	0.057
	1	5 (1.0)	0 (0.0)	6 (0.8)	2 (0.4)	11 (0.6)	33 (1.5)	0 (0.0)	0 (0.0)	
Pheniramine_b (%)	0	494 (99.8)	65 (100.0)	712 (99.3)	543 (99.8)	1699 (99.9)	2153 (100.0)	143 (100.0)	51 (100.0)	0.013
	1	1 (0.2)	0 (0.0)	5 (0.7)	1 (0.2)	2 (0.1)	0 (0.0)	0 (0.0)	0 (0.0)	
Phenobarbital_b (%)	0	495 (100.0)	58 (89.2)	715 (99.7)	542 (99.6)	1685 (99.1)	2150 (99.9)	143 (100.0)	51 (100.0)	<0.001
	1	0 (0.0)	7 (10.8)	2 (0.3)	2 (0.4)	16 (0.9)	3 (0.1)	0 (0.0)	0 (0.0)	
Phentermine_b (%)	0	492 (99.4)	65 (100.0)	714 (99.6)	538 (98.9)	1696 (99.7)	2145 (99.6)	142 (99.3)	49 (96.1)	0.006
	1	3 (0.6)	0 (0.0)	3 (0.4)	6 (1.1)	5 (0.3)	8 (0.4)	1 (0.7)	2 (3.9)	
Phenylephrine_b (%)	0	490 (99.0)	65 (100.0)	712 (99.3)	541 (99.4)	1672 (98.3)	1600 (74.3)	141 (98.6)	51 (100.0)	<0.001
	1	5 (1.0)	0 (0.0)	5 (0.7)	3 (0.6)	29 (1.7)	553 (25.7)	2 (1.4)	0 (0.0)	
Phenytoin_b (%)	0	491 (99.2)	65 (100.0)	712 (99.3)	543 (99.8)	1677 (98.6)	2141 (99.4)	142 (99.3)	51 (100.0)	0.067
	1	4 (0.8)	0 (0.0)	5 (0.7)	1 (0.2)	24 (1.4)	12 (0.6)	1 (0.7)	0 (0.0)	
Pimozide_b (%)	0	495 (100.0)	65 (100.0)	716 (99.9)	544 (100.0)	1700 (99.9)	2152 (100.0)	143 (100.0)	51 (100.0)	0.971
	1	0 (0.0)	0 (0.0)	1 (0.1)	0 (0.0)	1 (0.1)	1 (0.0)	0 (0.0)	0 (0.0)	
Pramipexole_b (%)	0	494 (99.8)	65 (100.0)	715 (99.7)	542 (99.6)	1692 (99.5)	2145 (99.6)	142 (99.3)	50 (98.0)	0.648
	1	1 (0.2)	0 (0.0)	2 (0.3)	2 (0.4)	9 (0.5)	8 (0.4)	1 (0.7)	1 (2.0)	
Pregabalin_b (%)	0	487 (98.4)	64 (98.5)	700 (97.6)	530 (97.4)	1672 (98.3)	2108 (97.9)	136 (95.1)	48 (94.1)	0.11
	1	8 (1.6)	1 (1.5)	17 (2.4)	14 (2.6)	29 (1.7)	45 (2.1)	7 (4.9)	3 (5.9)	
Primidone_b (%)	0	495 (100.0)	65 (100.0)	716 (99.9)	543 (99.8)	1700 (99.9)	2152 (100.0)	143 (100.0)	51 (100.0)	0.942
	1	0 (0.0)	0 (0.0)	1 (0.1)	1 (0.2)	1 (0.1)	1 (0.0)	0 (0.0)	0 (0.0)	
Promazine_b (%)	0	494 (99.8)	65 (100.0)	710 (99.0)	541 (99.4)	1689 (99.3)	2149 (99.8)	141 (98.6)	51 (100.0)	0.073
	1	1 (0.2)	0 (0.0)	7 (1.0)	3 (0.6)	12 (0.7)	4 (0.2)	2 (1.4)	0 (0.0)	
Promethazine_b (%)	0	491 (99.2)	64 (98.5)	705 (98.3)	538 (98.9)	1657 (97.4)	2057 (95.5)	140 (97.9)	51 (100.0)	<0.001
	1	4 (0.8)	1 (1.5)	12 (1.7)	6 (1.1)	44 (2.6)	96 (4.5)	3 (2.1)	0 (0.0)	
Propoxyphene_b (%)	0	494 (99.8)	64 (98.5)	715 (99.7)	544 (100.0)	1700 (99.9)	2150 (99.9)	143 (100.0)	51 (100.0)	0.089
	1	1 (0.2)	1 (1.5)	2 (0.3)	0 (0.0)	1 (0.1)	3 (0.1)	0 (0.0)	0 (0.0)	
Protriptyline_b (%)	0	495 (100.0)	65 (100.0)	716 (99.9)	544 (100.0)	1700 (99.9)	2153 (100.0)	143 (100.0)	51 (100.0)	0.8
	1	0 (0.0)	0 (0.0)	1 (0.1)	0 (0.0)	1 (0.1)	0 (0.0)	0 (0.0)	0 (0.0)	
Pseudoephedrine_b (%)	0	492 (99.4)	65 (100.0)	713 (99.4)	539 (99.1)	1691 (99.4)	2133 (99.1)	142 (99.3)	50 (98.0)	0.81
	1	3 (0.6)	0 (0.0)	4 (0.6)	5 (0.9)	10 (0.6)	20 (0.9)	1 (0.7)	1 (2.0)	
Quetiapine_b (%)	0	466 (94.1)	61 (93.8)	637 (88.8)	505 (92.8)	1566 (92.1)	2046 (95.0)	133 (93.0)	49 (96.1)	<0.001
	1	29 (5.9)	4 (6.2)	80 (11.2)	39 (7.2)	135 (7.9)	107 (5.0)	10 (7.0)	2 (3.9)	
Quinidine_b (%)	0	495 (100.0)	65 (100.0)	717 (100.0)	543 (99.8)	1700 (99.9)	2153 (100.0)	143 (100.0)	51 (100.0)	0.645
	1	0 (0.0)	0 (0.0)	0 (0.0)	1 (0.2)	1 (0.1)	0 (0.0)	0 (0.0)	0 (0.0)	
Remifentanil_b (%)	0	494 (99.8)	65 (100.0)	717 (100.0)	543 (99.8)	1701 (100.0)	2130 (98.9)	143 (100.0)	51 (100.0)	<0.001
	1	1 (0.2)	0 (0.0)	0 (0.0)	1 (0.2)	0 (0.0)	23 (1.1)	0 (0.0)	0 (0.0)	
Riboflavin_b (%)	0	495 (100.0)	65 (100.0)	712 (99.3)	543 (99.8)	1699 (99.9)	2150 (99.9)	143 (100.0)	51 (100.0)	0.098
	1	0 (0.0)	0 (0.0)	5 (0.7)	1 (0.2)	2 (0.1)	3 (0.1)	0 (0.0)	0 (0.0)	
Risperidone_b (%)	0	489 (98.8)	64 (98.5)	675 (94.1)	536 (98.5)	1630 (95.8)	2111 (98.0)	134 (93.7)	49 (96.1)	<0.001
	1	6 (1.2)	1 (1.5)	42 (5.9)	8 (1.5)	71 (4.2)	42 (2.0)	9 (6.3)	2 (3.9)	
Rizatriptan_b (%)	0	488 (98.6)	64 (98.5)	700 (97.6)	538 (98.9)	1690 (99.4)	2138 (99.3)	140 (97.9)	51 (100.0)	0.004
	1	7 (1.4)	1 (1.5)	17 (2.4)	6 (1.1)	11 (0.6)	15 (0.7)	3 (2.1)	0 (0.0)	
Ropinirole_b (%)	0	491 (99.2)	62 (95.4)	708 (98.7)	537 (98.7)	1686 (99.1)	2135 (99.2)	138 (96.5)	50 (98.0)	0.009
	1	4 (0.8)	3 (4.6)	9 (1.3)	7 (1.3)	15 (0.9)	18 (0.8)	5 (3.5)	1 (2.0)	
Ropivacaine_b (%)	0	495 (100.0)	65 (100.0)	713 (99.4)	543 (99.8)	1699 (99.9)	1963 (91.2)	142 (99.3)	51 (100.0)	<0.001
	1	0 (0.0)	0 (0.0)	4 (0.6)	1 (0.2)	2 (0.1)	190 (8.8)	1 (0.7)	0 (0.0)	
Rufinamide_b (%)	0	495 (100.0)	65 (100.0)	717 (100.0)	544 (100.0)	1700 (99.9)	2153 (100.0)	143 (100.0)	51 (100.0)	0.931
	1	0 (0.0)	0 (0.0)	0 (0.0)	0 (0.0)	1 (0.1)	0 (0.0)	0 (0.0)	0 (0.0)	
Salicylic Acid_b (%)	0	495 (100.0)	65 (100.0)	714 (99.6)	543 (99.8)	1698 (99.8)	2152 (100.0)	143 (100.0)	51 (100.0)	0.451
	1	0 (0.0)	0 (0.0)	3 (0.4)	1 (0.2)	3 (0.2)	1 (0.0)	0 (0.0)	0 (0.0)	
Scopolamine_b (%)	0	493 (99.6)	65 (100.0)	716 (99.9)	543 (99.8)	1699 (99.9)	2001 (92.9)	143 (100.0)	51 (100.0)	<0.001
	1	2 (0.4)	0 (0.0)	1 (0.1)	1 (0.2)	2 (0.1)	152 (7.1)	0 (0.0)	0 (0.0)	
Sertraline_b (%)	0	429 (86.7)	59 (90.8)	597 (83.3)	500 (91.9)	1475 (86.7)	1955 (90.8)	133 (93.0)	44 (86.3)	<0.001
	1	66 (13.3)	6 (9.2)	120 (16.7)	44 (8.1)	226 (13.3)	198 (9.2)	10 (7.0)	7 (13.7)	
Sodium Bicarbonate_b (%)	0	495 (100.0)	65 (100.0)	713 (99.4)	543 (99.8)	1700 (99.9)	2151 (99.9)	143 (100.0)	51 (100.0)	0.117
	1	0 (0.0)	0 (0.0)	4 (0.6)	1 (0.2)	1 (0.1)	2 (0.1)	0 (0.0)	0 (0.0)	
Sodium Oxybate_b (%)	0	495 (100.0)	65 (100.0)	717 (100.0)	544 (100.0)	1700 (99.9)	2153 (100.0)	143 (100.0)	51 (100.0)	0.931
	1	0 (0.0)	0 (0.0)	0 (0.0)	0 (0.0)	1 (0.1)	0 (0.0)	0 (0.0)	0 (0.0)	
Sufentanil_b (%)	0	495 (100.0)	65 (100.0)	717 (100.0)	544 (100.0)	1701 (100.0)	2152 (100.0)	143 (100.0)	51 (100.0)	0.973
	1	0 (0.0)	0 (0.0)	0 (0.0)	0 (0.0)	0 (0.0)	1 (0.0)	0 (0.0)	0 (0.0)	
Sumatriptan_b (%)	0	477 (96.4)	64 (98.5)	683 (95.3)	527 (96.9)	1632 (95.9)	2094 (97.3)	140 (97.9)	48 (94.1)	0.128
	1	18 (3.6)	1 (1.5)	34 (4.7)	17 (3.1)	69 (4.1)	59 (2.7)	3 (2.1)	3 (5.9)	
Suvorexant_b (%)	0	495 (100.0)	65 (100.0)	717 (100.0)	543 (99.8)	1700 (99.9)	2153 (100.0)	142 (99.3)	51 (100.0)	0.031
	1	0 (0.0)	0 (0.0)	0 (0.0)	1 (0.2)	1 (0.1)	0 (0.0)	1 (0.7)	0 (0.0)	
Tapentadol_b (%)	0	495 (100.0)	65 (100.0)	715 (99.7)	544 (100.0)	1701 (100.0)	2151 (99.9)	143 (100.0)	51 (100.0)	0.438
	1	0 (0.0)	0 (0.0)	2 (0.3)	0 (0.0)	0 (0.0)	2 (0.1)	0 (0.0)	0 (0.0)	
Temazepam_b (%)	0	495 (100.0)	65 (100.0)	717 (100.0)	544 (100.0)	1701 (100.0)	2152 (100.0)	0 (0.0)	51 (100.0)	<0.001
	1	0 (0.0)	0 (0.0)	0 (0.0)	0 (0.0)	0 (0.0)	1 (0.0)	143 (100.0)	0 (0.0)	
Thiamine_b (%)	0	490 (99.0)	50 (76.9)	712 (99.3)	536 (98.5)	1539 (90.5)	2125 (98.7)	142 (99.3)	51 (100.0)	<0.001
	1	5 (1.0)	15 (23.1)	5 (0.7)	8 (1.5)	162 (9.5)	28 (1.3)	1 (0.7)	0 (0.0)	
Thiothixene_b (%)	0	495 (100.0)	65 (100.0)	716 (99.9)	544 (100.0)	1701 (100.0)	2152 (100.0)	143 (100.0)	51 (100.0)	0.84
	1	0 (0.0)	0 (0.0)	1 (0.1)	0 (0.0)	0 (0.0)	1 (0.0)	0 (0.0)	0 (0.0)	
Tiagabine_b (%)	0	494 (99.8)	65 (100.0)	716 (99.9)	544 (100.0)	1701 (100.0)	2152 (100.0)	143 (100.0)	51 (100.0)	0.71
	1	1 (0.2)	0 (0.0)	1 (0.1)	0 (0.0)	0 (0.0)	1 (0.0)	0 (0.0)	0 (0.0)	
Topiramate_b (%)	0	470 (94.9)	62 (95.4)	663 (92.5)	501 (92.1)	1604 (94.3)	2076 (96.4)	132 (92.3)	49 (96.1)	<0.001
	1	25 (5.1)	3 (4.6)	54 (7.5)	43 (7.9)	97 (5.7)	77 (3.6)	11 (7.7)	2 (3.9)	
Tramadol_b (%)	0	460 (92.9)	61 (93.8)	665 (92.7)	498 (91.5)	1602 (94.2)	1981 (92.0)	128 (89.5)	45 (88.2)	0.104
	1	35 (7.1)	4 (6.2)	52 (7.3)	46 (8.5)	99 (5.8)	172 (8.0)	15 (10.5)	6 (11.8)	
Tranylcypromine_b (%)	0	495 (100.0)	65 (100.0)	717 (100.0)	544 (100.0)	1700 (99.9)	2153 (100.0)	143 (100.0)	51 (100.0)	0.931
	1	0 (0.0)	0 (0.0)	0 (0.0)	0 (0.0)	1 (0.1)	0 (0.0)	0 (0.0)	0 (0.0)	
Trazodone_b (%)	0	451 (91.1)	46 (70.8)	618 (86.2)	477 (87.7)	1479 (86.9)	1979 (91.9)	115 (80.4)	46 (90.2)	<0.001
	1	44 (8.9)	19 (29.2)	99 (13.8)	67 (12.3)	222 (13.1)	174 (8.1)	28 (19.6)	5 (9.8)	
Triazolam_b (%)	0	495 (100.0)	65 (100.0)	717 (100.0)	544 (100.0)	1701 (100.0)	2153 (100.0)	143 (100.0)	0 (0.0)	<0.001
	1	0 (0.0)	0 (0.0)	0 (0.0)	0 (0.0)	0 (0.0)	0 (0.0)	0 (0.0)	51 (100.0)	
Trifluoperazine_b (%)	0	495 (100.0)	65 (100.0)	716 (99.9)	544 (100.0)	1699 (99.9)	2153 (100.0)	143 (100.0)	51 (100.0)	0.741
	1	0 (0.0)	0 (0.0)	1 (0.1)	0 (0.0)	2 (0.1)	0 (0.0)	0 (0.0)	0 (0.0)	
Valproate_b (%)	0	494 (99.8)	65 (100.0)	715 (99.7)	544 (100.0)	1687 (99.2)	2150 (99.9)	143 (100.0)	51 (100.0)	0.015
	1	1 (0.2)	0 (0.0)	2 (0.3)	0 (0.0)	14 (0.8)	3 (0.1)	0 (0.0)	0 (0.0)	
Venlafaxine_b (%)	0	458 (92.5)	61 (93.8)	635 (88.6)	513 (94.3)	1566 (92.1)	2065 (95.9)	129 (90.2)	51 (100.0)	<0.001
	1	37 (7.5)	4 (6.2)	82 (11.4)	31 (5.7)	135 (7.9)	88 (4.1)	14 (9.8)	0 (0.0)	
Vilazodone_b (%)	0	492 (99.4)	65 (100.0)	715 (99.7)	543 (99.8)	1697 (99.8)	2148 (99.8)	142 (99.3)	50 (98.0)	0.307
	1	3 (0.6)	0 (0.0)	2 (0.3)	1 (0.2)	4 (0.2)	5 (0.2)	1 (0.7)	1 (2.0)	
Vitamin B6_b (%)	0	494 (99.8)	65 (100.0)	716 (99.9)	542 (99.6)	1689 (99.3)	2137 (99.3)	143 (100.0)	51 (100.0)	0.393
	1	1 (0.2)	0 (0.0)	1 (0.1)	2 (0.4)	12 (0.7)	16 (0.7)	0 (0.0)	0 (0.0)	
Vortioxetine_b (%)	0	494 (99.8)	64 (98.5)	714 (99.6)	541 (99.4)	1691 (99.4)	2149 (99.8)	139 (97.2)	51 (100.0)	0.001
	1	1 (0.2)	1 (1.5)	3 (0.4)	3 (0.6)	10 (0.6)	4 (0.2)	4 (2.8)	0 (0.0)	
Zaleplon_b (%)	0	494 (99.8)	65 (100.0)	716 (99.9)	543 (99.8)	1700 (99.9)	2151 (99.9)	141 (98.6)	51 (100.0)	0.011
	1	1 (0.2)	0 (0.0)	1 (0.1)	1 (0.2)	1 (0.1)	2 (0.1)	2 (1.4)	0 (0.0)	
Ziprasidone_b (%)	0	491 (99.2)	64 (98.5)	702 (97.9)	541 (99.4)	1674 (98.4)	2135 (99.2)	139 (97.2)	51 (100.0)	0.032
	1	4 (0.8)	1 (1.5)	15 (2.1)	3 (0.6)	27 (1.6)	18 (0.8)	4 (2.8)	0 (0.0)	
Zolmitriptan_b (%)	0	494 (99.8)	65 (100.0)	713 (99.4)	544 (100.0)	1701 (100.0)	2152 (100.0)	143 (100.0)	51 (100.0)	0.011
	1	1 (0.2)	0 (0.0)	4 (0.6)	0 (0.0)	0 (0.0)	1 (0.0)	0 (0.0)	0 (0.0)	
Zolpidem_b (%)	0	456 (92.1)	59 (90.8)	665 (92.7)	517 (95.0)	1598 (93.9)	2015 (93.6)	123 (86.0)	49 (96.1)	0.008
	1	39 (7.9)	6 (9.2)	52 (7.3)	27 (5.0)	103 (6.1)	138 (6.4)	20 (14.0)	2 (3.9)	
Zonisamide_b (%)	0	493 (99.6)	65 (100.0)	713 (99.4)	542 (99.6)	1690 (99.4)	2151 (99.9)	143 (100.0)	51 (100.0)	0.189
	1	2 (0.4)	0 (0.0)	4 (0.6)	2 (0.4)	11 (0.6)	2 (0.1)	0 (0.0)	0 (0.0)	
Doxazosin_b (%)	0	494 (99.8)	65 (100.0)	716 (99.9)	544 (100.0)	1700 (99.9)	2148 (99.8)	141 (98.6)	51 (100.0)	0.029
a	1	1 (0.2)	0 (0.0)	1 (0.1)	0 (0.0)	1 (0.1)	5 (0.2)	2 (1.4)	0 (0.0)	
Prazosin_b (%)	0	484 (97.8)	65 (100.0)	666 (92.9)	516 (94.9)	1610 (94.7)	2067 (96.0)	133 (93.0)	49 (96.1)	0.001
	1	11 (2.2)	0 (0.0)	51 (7.1)	28 (5.1)	91 (5.3)	86 (4.0)	10 (7.0)	2 (3.9)	
Terazosin_b (%)	0	494 (99.8)	65 (100.0)	714 (99.6)	542 (99.6)	1697 (99.8)	2141 (99.4)	142 (99.3)	51 (100.0)	0.796
	1	1 (0.2)	0 (0.0)	3 (0.4)	2 (0.4)	4 (0.2)	12 (0.6)	1 (0.7)	0 (0.0)	

## References

[1] Substance Abuse and Mental Health Services Administration (2014). Trauma-Informed Care in Behavioral Health Services. Treatment Improvement Protocol (TIP) Series 57. HHS Publication No. (SMA) 13-4801.

[2] Kessler R.C., Sonnega A., Bromet E., Hughes M., Nelson C.B. (1995). Posttraumatic stress disorder in the National Comorbidity Survey. Arch. Gen. Psychiatry.

[3] Benatti B., Ferrari S., Grancini B., Girone N., Briguglio M., Marazziti D., Mucci F., Dell’Osso L., Gambini O., Demartini B. (2020). Suicidal ideation and suicidal attempts in patients with obsessive-compulsive tic-related disorder vs obsessive-compulsive disorder: Results of a multicenter Italian study. CNS Spectr..

[4] Sareen J., Houlahan T., Cox B.J., Asmundson G.J. (2005). Anxiety disorders associated with suicidal ideation and suicide attempts in the National Comorbidity Survey. J. Nerv. Ment. Dis..

[5] Weber F.C., Norra C., Wetter T.C. (2020). Sleep disturbances and suicidality in posttraumatic stress disorder: An overview of the literature. Front. Psychiatry.

[6] Hagnell O., Lanke J., Rorsman B. (1981). Suicide rates in the Lundby study: Mental illness as a risk factor for suicide. Neuropsychobiology.

[7] Krysinska K., Lester D. (2010). Post-traumatic stress disorder and suicide risk: A systematic review. Arch. Suicide Res..

[8] Gradus J.L., Qin P., Lincoln A.K., Miller M., Lawler E., Sørensen H.T., Lash T.L. (2010). Posttraumatic stress disorder and completed suicide. Am. J. Epidemiol..

[9] Mendes D.D., Mello M.F., Ventura P., De Medeiros Passarela C., De Jesus Mari J. (2008). A systematic review on the effectiveness of cognitive behavioral therapy for posttraumatic stress disorder. Int. J. Psychiatry Med..

[10] Kozaric-Kovacic D. (2008). Psychopharmacotherapy of posttraumatic stress disorder. Croat. Med. J..

[11] Nappi C.M., Drummond S.P.A., Hall J.M.H. (2012). Treating nightmares and insomnia in posttraumatic stress disorder: A review of current evidence. Neuropharmacology.

[12] De Moraes Costa G., Zanatta F.B., Ziegelmann P.K., Barros A.J.S., Mello C.F. (2020). Pharmacological treatments for adults with post-traumatic stress disorder: A network meta-analysis of comparative efficacy and acceptability. J. Psychiatr. Res..

[13] Adetunji B., Mathews M., Williams A., Budur K., Mathews M., Mahmud J., Osinowo T. (2005). Use of antipsychotics in the treatment of post-traumatic stress disorder. Psychiatry (Edgmont).

[14] Berardis D.D., Marini S., Serroni N., Iasevoli F., Tomasetti C., de Bartolomeis A., Mazza M., Tempesta D., Valchera A., Fornaro M. (2015). Targeting the noradrenergic system in posttraumatic stress disorder: A systematic review and meta-analysis of prazosin trials. Curr. Drug Targets.

[15] McIntosh B., Clark M., Spry C. (2011). CADTH Rapid Response Reports. Benzodiazepines in Older Adults: A Review of Clinical Effectiveness, Cost-Effectiveness, and Guidelines.

[16] Lund B.C., Bernardy N.C., Vaughan-Sarrazin M., Alexander B., Friedman M.J. (2013). Patient and facility characteristics associated with benzodiazepine prescribing for veterans with PTSD. Psychiatr. Serv..

[17] Guina J., Rossetter S.R., De R.B., Nahhas R.W., Welton R.S. (2015). Benzodiazepines for PTSD: A Systematic Review and Meta-Analysis. J. Psychiatr. Pract..

[18] Harpaz-Rotem I., Rosenheck R.A., Mohamed S., Desai R.A. (2008). Pharmacologic treatment of posttraumatic stress disorder among privately insured Americans. Psychiatr. Serv..

[19] Lund B.C., Bernardy N.C., Alexander B., Friedman M.J. (2012). Declining benzodiazepine use in veterans with posttraumatic stress disorder. J. Clin. Psychiatry.

[20] Hawkins E.J., Malte C.A., Imel Z.E., Saxon A.J., Kivlahan D.R. (2012). Prevalence and trends of benzodiazepine use among Veterans Affairs patients with posttraumatic stress disorder, 2003–2010. Drug Alcohol Depend..

[21] Emilsson L., García-Albéniz X., Logan R.W., Caniglia E.C., Kalager M., Hernán M.A. (2018). Examining Bias in Studies of Statin Treatment and Survival in Patients with Cancer. JAMA Oncol..

[22] Danaei G., García Rodríguez L.A., Cantero O.F., Logan R.W., Hernán M.A. (2018). Electronic medical records can be used to emulate target trials of sustained treatment strategies. J. Clin. Epidemiol..

[23] Ihaka R., Gentleman R. (1996). R: A language for data analysis and graphics. J. Comput. Graph. Stat..

[24] Cato V., Holländare F., Nordenskjöld A., Sellin T. (2019). Association between benzodiazepines and suicide risk: A matched case-control study. BMC Psychiatry.

[25] Sanderson M., Bulloch A.G.M., Wang J., Williams K.G., Williamson T., Patten S.B. (2020). Predicting death by suicide following an emergency department visit for parasuicide with administrative health care system data and machine learning. EClinicalMedicine.

